# Piezo1-mediated mechanosensation in bone marrow macrophages promotes vascular niche regeneration after irradiation injury

**DOI:** 10.7150/thno.64963

**Published:** 2022-01-16

**Authors:** Xiaomei Zhang, Lijia Hou, Fengjie Li, Weiwei Zhang, Chun Wu, Lixin Xiang, Jiuxuan Li, Luping Zhou, Xiaojie Wang, Yang Xiang, Yanni Xiao, Shengwen Calvin Li, Li Chen, Qian Ran, Zhongjun Li

**Affiliations:** 1 Laboratory of Radiation Biology, Laboratory Medicine Center, Department of Blood Transfusion, The Second Affiliated Hospital, Army Military Medical University, Chongqing 400037, China; 2 111 Project Laboratory of Biomechanics and Tissue Repair, College of Bioengineering, Chongqing University, Chongqing 400044, China; 3 Neuro-oncology and Stem Cell Research Laboratory, CHOC Children's Research Institute, Children's Hospital of Orange County (CHOC); 1201 La Veta Ave., Orange, CA 92868-3874, USA; 4 Department of Neurology, University of California - Irvine School of Medicine, 200 S. Manchester Ave. Ste. 206, Orange, CA 92868, USA

**Keywords:** irradiation, Piezo1, macrophages, sinusoidal regeneration, hematopoietic reconstitution

## Abstract

**Background:** Irradiation disrupts the vascular niche where hematopoietic stem cells (HSCs) reside, causing delayed hematopoietic reconstruction. The subsequent recovery of sinusoidal vessels is key to vascular niche regeneration and a prerequisite for hematopoietic reconstruction. We hypothesize that resident bone marrow macrophages (BM-Mφs) are responsible for repairing the HSC niche upon irradiation injury.

**Methods:** We examined the survival and activation of BM-Mφs in C57BL/6 mice upon total body irradiation. After BM-Mφ depletion via injected clodronate-containing liposomes and irradiation injury, hematopoietic reconstruction and sinusoidal vascular regeneration were assessed with immunofluorescence and flow cytometry. Then enzyme-linked immunosorbent assay (ELISA) and flow cytometry were performed to analyze the contribution of VEGF-A released by BM-Mφs to the vascular restructuring of the HSC niche. VEGF-A-mediated signal transduction was assessed with transcriptome sequencing, flow cytometry, and pharmacology (agonists and antagonists) to determine the molecular mechanisms of Piezo1-mediated responses to structural changes in the HSC niche.

**Results:** The depletion of BM-Mφs aggravated the post-irradiation injury, delaying the recovery of sinusoidal endothelial cells and HSCs. A fraction of the BM-Mφ population persisted after irradiation, with residual BM-Mφ exhibiting an activated M2-like phenotype. The expression of VEGF-A, which is essential for sinusoidal regeneration, was upregulated in BM-Mφs post-irradiation, especially CD206^+^ BM-Mφs. The expression of mechanosensory ion channel Piezo1, a response to mechanical environmental changes induced by bone marrow ablation, was upregulated in BM-Mφs, especially CD206^+^ BM-Mφs. Piezo1 upregulation was mediated by the effects of irradiation, the activation of Piezo1 itself, and the M2-like polarization induced by the phagocytosis of apoptotic cells. Piezo1 activation was associated with increased expression of VEGF-A and increased accumulation of NFATC1, NFATC2, and HIF-1α. The Piezo1-mediated upregulation in VEGF-A was suppressed by inhibiting the calcineurin/NFAT/HIF-1α signaling pathway.

**Conclusion:** These findings reveal that BM-Mφs play a critical role in promoting vascular niche regeneration by sensing and responding to structural changes after irradiation injury, offering a potential target for therapeutic efforts to enhance hematopoietic reconstruction.

## Introduction

Hematopoietic transplantation after irradiation or chemotherapy is the primary treatment for leukemia and other malignant and non-malignant diseases 1-3. However, irradiation or chemotherapy destroys the local microenvironment, including the hematopoietic stem cell (HSC) niche where HSCs reside, leading to delayed or failed hematopoietic reconstitution. The HSC niche consists of bone marrow stromal components containing stromal cells, sympathetic nerve fibers, endothelial cells (ECs), and resident bone marrow macrophages (BM-Mφs), which are the mature progeny of HSCs. The HSC niche tightly regulates the self-renewal and differentiation of HSCs to mature progeny in the BM 4. Accumulated evidence supports the regulation of HSC homeostasis by resident BM-Mφs, which promote the HSC retention via crosstalk with osteoblast and mesenchymal stem cells (MSCs) 5, 6. BM-Mφs with specific phenotypes can directly interact with HSCs; for example, CD234^+^-expressing Mφs promote HSC quiescence (i.e., dormancy) by activating CD82 expressed on the HSC surface 7. At the same time, α-smooth muscle actin-expressing Mφs maintain the pool of quiescent HSCs by secreting prostaglandin E2 (PGE2) to decrease levels of reactive oxygen species (ROS) 8. Depleting BM-Mφs induces the egress of HSCs into the blood, simultaneously increasing HSC proliferation and maintaining steady-state levels of quiescent HSCs in response to interferon-gamma (IFNγ) 9. BM-Mφs are relatively resistant to apoptosis during irradiation or chemotherapy-induced bone marrow hematopoietic injury 7. Residual BM-Mφs that survive irradiation or chemotherapy can self-repopulate via *in situ* autonomous proliferation to promote long-term HSC engraftment and hematopoietic reconstitution 10. Although it has been suggested that Mφs guide the homing of transplanted HSCs to a vascular niche and subsequent retention in the BM 11, the underlying mechanisms of Mφ-mediated hematopoietic reconstitution post-irradiation-induced bone marrow hematopoietic injury remain obscure. This study was designed to elucidate the role of BM-Mφ in hematopoietic reconstitution after HSC niche injury.

HSCs are often widely distributed in vascular niches. About 80% are located near the sinusoids, which are irregular tubular spaces for the passage of blood that supplant capillaries and venules in the bone marrow, a.k.a., the BM microvascular system 12. Bone marrow sinusoidal endothelium damage caused by chemotherapy or irradiation is often accompanied by destruction of the sinusoidal vasculature 13. The infusion of vascular endothelial/progenitor cells allows for recovery of the sinusoidal vasculature, leading to hematopoietic reconstruction 14. Disrupting the signaling of vascular endothelial growth factor-A (VEGF-A)/VEGF receptor 2 (VEGFR2) impairs recovery of the sinusoidal vasculature, thereby hindering hematopoietic reconstruction 15. Thus, repairing sinusoids is a critical step for bone marrow hematopoietic reconstruction. Studies have shown that Mφs play a vital role in the processes of vascular repair and angiogenesis required for tissue repair. Mφs mediate the repair of vascular ruptures through direct physical adhesion and mechanical traction, which may involve Piezo1 and Piezo2 16 [Ardem Patapoutian's work was recognized by The Nobel Prize in Physiology or Medicine 2021] and also indirectly through the secretion of pro-angiogenic factors, including VEGF-A 17. It is still unclear whether Mφs play a role in the regeneration of bone marrow sinusoids, which is a precondition for hematopoietic reconstruction.

Here, we identified a critical role for BM-Mφs in hematopoietic reconstitution through the promotion of sinusoidal regeneration post-irradiation injury. Mechanistically, we found that the VEGF-A expression was upregulated in Mφs in response to bone marrow ablation-causing mechanical stretching through the Piezo1 ion channel during irradiation injury.

## Methods

### Mice

All animal experiments were performed on 6-8-week-old C57BL/6 mice from Beijing HFK Bioscience Co., Ltd. Mice were maintained under specific pathogen-free conditions and fed standard mouse chow and water in the Animal Experimental Center of the Third Military Medical University. All procedures were approved by the Laboratory Animal Welfare and Ethics Committee of the Third Military Medical University.

For irradiation treatment, mice were exposed to Co-60 γ-rays and received a single dose of 5 Gy or 7.5 Gy total body irradiation at a rate of 0.69 Gy/min. For macrophage depletion, mice were injected with 200 μL clodronate-containing liposomes (Clo-lip, Yeasen, China) by tail vein one day before irradiation treatment. Control mice were injected with 200 μL PBS-containing liposomes (PBS-lip, Yeasen, China).

### Cell culture and treatment

For the generation of bone marrow-derived macrophages (BMDMs *in vitro*, BM-Mφs *in vivo*), mice were euthanized and rinsed in 75% ethanol for 5 min. The tibia and femur bones were isolated. After the ends were cut off, bones were flushed gently with 10 mL of sterile RPMI medium (HyClone, USA) with 10% fetal bovine serum (FBS, HyClone, USA), 100 U/mL penicillin, and 0.1 mg/mL streptomycin (HyClone, USA), using a 27½ gauge needle. Then 5×10^6^ bone marrow cells were seeded into 10 cm^2^ tissue culture plates and cultured in 10 mL RPMI medium containing murine macrophage colony-stimulating factor (M-CSF) (50 ng/mL) (315-02-100, PeproTech, USA), 10% FBS, 100 U/mL penicillin and 0.1 mg/mL streptomycin, at 37 °C in an atmosphere containing 5% CO_2_. The medium was changed on the fourth day. On the 7th day, the BMDMs were gently scraped off plates, counted, and seeded in new plates for experimentation. Murine RAW264.7 macrophages were cultured in DMEM medium (HyClone, USA) supplemented with 10% FBS, 100 U/mL penicillin and 0.1 mg/mL streptomycin.

To study the phagocytosis of apoptotic cells by BMDMs, mice were irradiated with 9 Gy. After 4 h, the apoptotic bone marrow cells were collected and labeled with DiI (C1036, Beyotime, China). BMDMs were incubated in 6-well plates with 5×10^6^ DiI-labeled apoptotic bone marrow cells. The unphagocytosed cells were removed by washing with PBS after 1 h. After 24 h, BMDMs were used for immunofluorescence or total RNA extraction.

To study the effect of mechanical stretching, BMDMs or RAW264.7 cells were treated with Yoda1 (S6678, Selleck, USA), with or without pretreatment with inhibitors for 30 min. The inhibitors included cyclosporin A (CsA, S2286, Selleck, USA), FK506 (HY-13756, MCE, USA), echinomycin (Ecn, HY-106101, MCE, USA), GsMTx4 (P1205, Selleck, USA), BAPTA-AM (S7534, Selleck, USA) and RN-1734 (S8107, Selleck, USA). In some experiments, BMDMs were treated with GSK1016790A (S2650, Selleck, USA).

### Blood cell count

Mice were anesthetized with 2% isoflurane, and anesthesia was maintained by continuous administration of 1% isoflurane using an isoflurane anesthesia machine (Tabletop, Harvard Corp., USA). The microhematocrit tube was inserted through the conjunctiva and into the orbital sinus by rotating the tube 18. Blood was collected into tubes containing ethylene diamine tetra-acetic acid (EDTA). After the required amount of blood was obtained, the tube was withdrawn, and bleeding was stopped by orbital pressure on the eye with a sterile cotton swab. An automatic animal blood cell analyzer (Prandre XFA6030, Nanjing Prande Medical Equipment Co., Ltd., China) was used to count the cells.

### Flow cytometry

The bone marrow was harvested by flushing the femur with 1 mL digestion buffer containing 2 mg/mL collagenase I and incubated at 37 °C for 30 min, with vortexing every 10 min. The red blood cells (RBCs) were lysed using ACK Lysis Buffer (C3702, Beyotime, China) for 5 min. Bone marrow nucleated cells (BMNCs) were then filtered through a 70-μm filter and stained for 30 min at 4 °C in the dark using the following antibodies: APC-Cy7 CD11b (101226), APC F4/80 (123116), FITC CD11c (117306), PE CD206 (141706), AF700 Lineage Cocktail (133313), APC c-Kit (105812), PE-Cy-7 Sca-1 (108114), PB CD48 (103418), PE CD150 (115904), APC-Cy7 Ter119 (116223), AF700 CD45 (103128), PB CD31 (102422), and PE-Cy7 CD105 (120410), all purchased from BioLegend (USA). 7-AAD (559925, B.D., USA) staining was used to exclude dead cells. For Piezo1 and VEGF-A staining, cells were fixed and permeabilized after surface antigen staining using Intracellular Staining Perm Wash Buffer (421002, BioLegend, USA) according to the manufacturer's protocol. Cells were stained with rabbit polyclonal antibody to Piezo1 (ab128245, Abcam, USA) or rabbit monoclonal antibody to VEGF-A (ab52917, Abcam, USA) for 30 min at 4 °C. Cells then were incubated with Alexa Fluor 488-conjugated donkey anti-rabbit secondary antibody (1:2000, ab150153, Abcam, USA) for 30 min at 4 °C. Ten thousand beads (424902, BioLegend, USA) were added to count cells. Flow cytometry was performed on the Gallios Flow Cytometer (Beckman, USA). FlowJo Software Version 10 (FlowJo LLC, USA) was used for subsequent analyses.

### Cell sorting and RNA sequencing

Cell sorting was performed using a FACS AriaIII cell sorter (B.D., USA). CD45^+^F480^+^ BM-Mφs were sorted from mice with non-irradiation or 3 or 7 days after irradiation. The total RNA of sorted cells was extracted using TRIzol reagent (15596-026, Ambion, USA). Total RNA concentration and purity were detected with Agilent 2100. cDNA libraries were sequenced to RNA-Seq using the BGISEQ platform (Shenzhen Huada Gene Science and Technology Service Co., Ltd., Shenzhen, China). The raw data was filtered to remove the adapter sequences, low-quality reads, higher N rate sequences (> 5%), and sequences that were too short. The remaining high-quality reads were mapped to the reference GRCm38.p6 using HISAT software. Fragments per kilobase of transcript per million mapped fragments (FPKM) represent each gene's expression quantity. False discovery rate (FDR) < 0.05 and |Log_2_FC (fold change) | ≥ 1 were used as the threshold for judging the significance of the difference in gene expression. Gene ontology (GO) and functional enrichment analysis were conducted on all differentially expressed genes (DEGs) identified using the Dr. Tom analysis system (https://biosys.bgi.com, Shenzhen Huada Gene Science and Technology Service Co., Ltd., Shenzhen, China). The RNA-Seq data were deposited in NCBI's Sequence Read Archive (SRA) with accession number SRP301166.

### Immunofluorescence

Femurs were fixed in 4% paraformaldehyde (PFA) for 24 h, decalcified in 12% EDTA for 3 weeks, dehydrated in 30% sucrose for 2 days, then embedded in optimal cutting temperature (OCT) medium (Tissue-Tek, Japan) and sliced into 10-μm cryosections. BMDMs were fixed in 4% PFA for 20 min. BMDMs and sections were permeabilized with 0.25% Triton X-100 for 5 min, blocked with 2% bovine serum albumin (BSA) for 1 h, and stained with rat monoclonal antibody to CD105 (1:100, 120401, BioLegend, USA), rat monoclonal antibody to F4/80 (1:200, ab6640, Abcam, USA), or rabbit polyclonal antibody to CD206 (1:200, ab64693, Abcam, USA) overnight at 4 °C. The secondary antibodies used were Alexa Fluor 488-conjugated donkey anti-rat (ab150153) or anti-rabbit (ab150073) (1:500, Abcam, USA). Nuclei were labeled with DAPI (C1002, Beyotime, China). Images were acquired with an inverted fluorescence microscope (Olympus IX71, Tokyo, Japan) or Zeiss LSM 510 laser scanning microscope (Zeiss, Jena, Germany).

### Enzyme-linked Immunosorbent Assay (ELISA)

Bone marrow levels of VEGF-A were determined as follows. Both tibias and femurs were isolated, and the ends of the bones were cut off. The bones were placed in a 0.5-mL Eppendorf tube cut open at the bottom and nestled inside a 1.5-mL Eppendorf tube. The bone marrow was spun out by brief centrifugation (from 0 rpm to 10,000 rpm, 9 s). Then, the bone marrow from both femurs and tibias was lysed with 400 μL cell lysis buffer for western blotting and immunoprecipitation (P0013, Beyotime, China). The supernatant was collected for ELISA. To assess VEGF-A expression of BMDMs or RAW264.7 cells, 24 h after Yoda1 treatment with or without inhibitor pretreatment for 30 minutes, the cell medium was centrifuged at 13,000×*g* for 10 min to remove cellular debris. Concentrations of VEGF-A were measured using the Mouse VEGF-A ELISA Kit (EK0541, Boster, China), according to the manufacturer's instructions.

### Real-time polymerase chain reaction (RT-PCR)

Total RNA was isolated from cells using TRIzol (15596-026, Ambion, USA). 1 μg total RNA was reverse transcribed into cDNA using the PrimeScript™ RT reagent Kit with gDNA Eraser (RR047A, Takala, Dalian, China) according to the manufacturer's protocol. RT-PCR was conducted using TB Green® Premix Ex Taq™ II (RR820A, Takara, Dalian, China). The reaction protocol was as follows: heating for 30 s at 95 °C, followed by 40 cycles of amplification (5 s at 95 °C and 30 s at 60 °C). Fold changes in mRNA expression were calculated using ΔΔCt analysis 19. Primer sequences are provided in Table 1.

### Western blot analyses

Cells were lysed over 30 min with RIPA buffer (P0013B, Beyotime, China) containing a protease inhibitor cocktail on ice to harvest total protein. The protein concentration of cell lysate was measured with a BCA protein quantification kit (PC0020, Solarbio, China), according to the manufacturer's protocol. Equal amounts of protein were separated by 5-10% SDS-PAGE and electrophoretically transferred to a polyvinylidene difluoride (PVDF) membrane (BD Biosciences, USA). After blocking for 1 h in Western Blocking Buffer (P0023B, Beyotime, China) at room temperature, membranes were incubated with rabbit antibodies: anti-NFATC1 (1:1000, #8032, Cell Signaling Technology, USA), anti-NFATC3 (1:1000, #4998, Cell Signaling Technology, USA), anti-HIF-1α (1:1000, ab179483, Abcam, USA), and anti-GAPDH (1:5000, K106389P, Solarbio, China) at 4 ºC overnight. Membranes were incubated with appropriate horseradish peroxidase-conjugated goat anti-rabbit secondary antibody (1:1000, Beyotime, China) for 2 h at room temperature. Finally, signal was detected with SuperSignal West Femto Maximum Sensitivity Substrate (34095, Thermo Scientific, USA) and visualized by VersaDoc (Bio-Rad, USA).

### Statistical Analysis

Data are presented as mean ± standard deviation (SD) from at least three independent experiments unless otherwise stated. Statistical analysis was conducted with GraphPad Prism software (version 6, GraphPad Software). A two-tailed unpaired t-test (parametric data) or Mann-Whitney test (nonparametric data) was used for comparisons between two groups. Comparisons between multiple groups were performed with one-way analysis of variance (ANOVA) with Tukey test (parametric data) or Kruskal-Wallis test (nonparametric data). The value of *P* < 0.05 was considered to be statistically significant (*, *P* < 0.05; **, *P* < 0.01; ***, *P* < 0.001; ****, *P* < 0.0001; ns, not significant).

## Results

### BM-Mφ persistence and activation after irradiation

**Figure 1:**
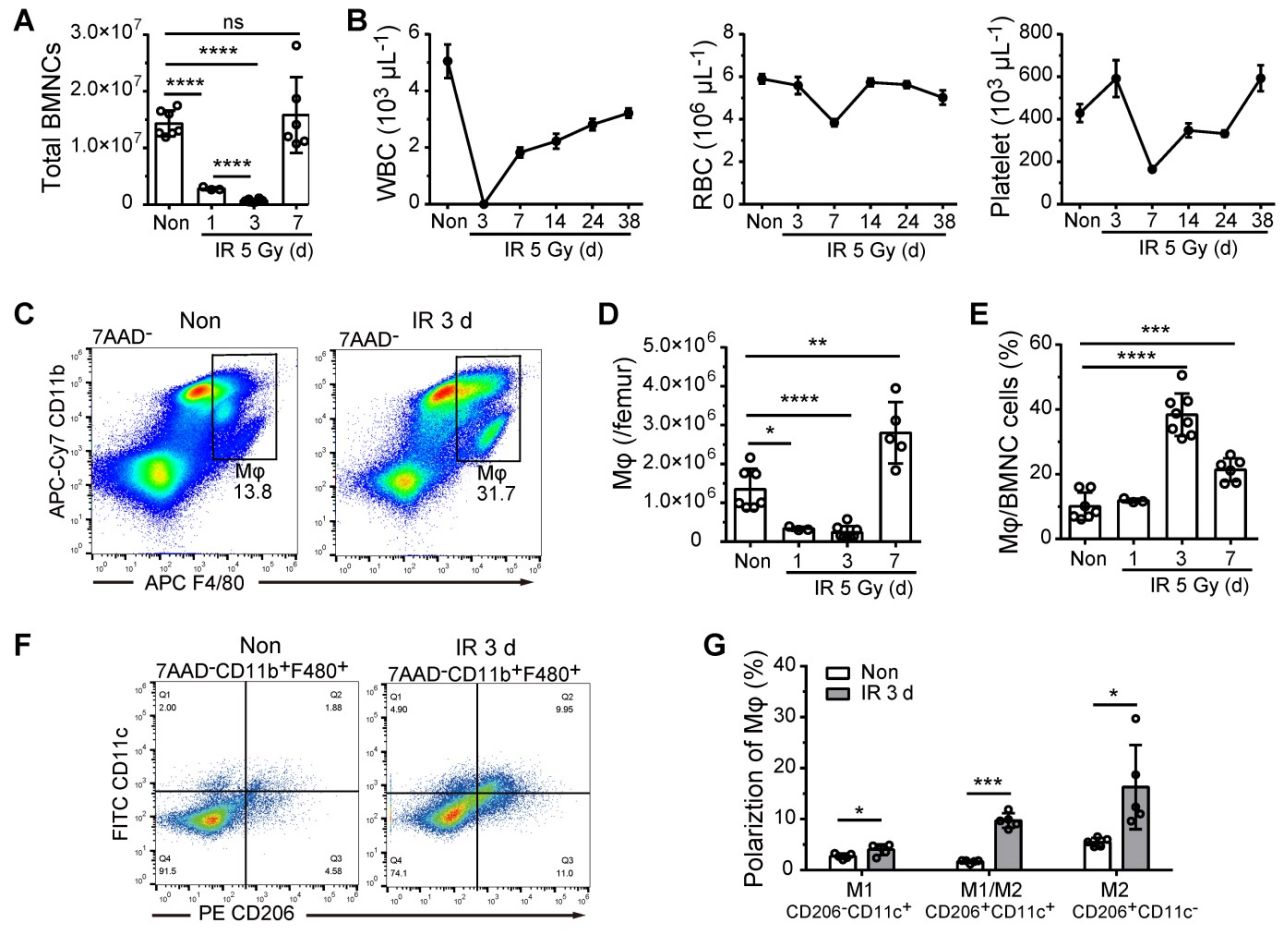
**BM-Mφs persist and are activated upon irradiation injury. (A)** Number of bone marrow nucleated cells (BMNCs) per femur after 5-Gy irradiation (IR) (n = 3-8 mice, Tukey test). **(B)** White blood cell (WBC), red blood cell (RBC), and platelet counts were measured in peripheral blood samples after 5-Gy irradiation (n = 3-8 mice).** (C)** Representative flow cytometry analysis of bone marrow-resident macrophages (BM-Mφs) (CD11b^+^F4/80^+^) without irradiation (Non) vs. 3 days after irradiation (IR 3d).** (D)** Number of BM-Mφs per femur and** (E)** percentage of BM-Mφs in the BMNC population after 5-Gy irradiation (n = 3-8 mice, Tukey test). **(F)** Representative flow cytometry analysis of BM-Mφs activation and persistence of M1-like Mφs (CD11b^+^F4/80^+^CD206^-^CD11c^+^), M1/M2-like Mφs (CD11b^+^F4/80^+^CD206^+^CD11c^+^), and M2-like Mφs (CD11b^+^F4/80^+^CD206^+^CD11c^-^). **(G)** Percentage of polarized BM-Mφs among all BM-Mφs (n = 5 mice, t-test). Graphs show the mean ± SD of pooled data from at least two independent experiments. *P < 0.05; **P < 0.01; ***P < 0.001; ****P < 0.0001; ns, not significant.

To examine the role of BM-Mφs in hematopoietic regeneration, we irradiated C57BL/6 mice with a single 5-Gy dose of radiation to obtain a model of moderate hematopoietic injury, which allowed for the repair of BM hematopoiesis without hematopoietic transplantation. As shown in Figure 1A, in the first three days post-irradiation, BMNC levels decreased drastically to approximately 5% of the level observed in non-irradiated mice; however, the BMNC population had recovered almost completely by day 7. Numbers of white blood cells (WBCs), RBCs, and platelets in peripheral blood also decreased to a minimum and then began to recover within one-week post-irradiation (Figure 1B). Similarly, the BM-Mφ cell population (CD11b^+^F480^+^) was reduced to about 18% of the normal, with rapid recovery by day 7 (Figure 1C-D). Notably, on the third-day post-irradiation, when most of the BMNCs (not including RBCs and platelets) were ablated, the proportion of BM-Mφs in the BMNC population was significantly higher than that in non-irradiated bone marrow (38% vs. 10%, *P* < 0.0001) (Figure 1E). With the recovery of hematopoiesis, the proportion of BM-Mφs began to decline to 21% by day 7 (Figure 1E). These results indicate that BM-Mφs had more radiation resistance than other BMNCs and were a relatively large cell population in the BM hematopoietic microenvironment in the early stage of irradiation injury.

BM-Mφs are a heterogeneous population with the plasticity and versatility to respond to microenvironmental cues 20. The classic theory of M1/M2-type activation is widely applied to investigate the roles of BM-Mφs in developing and treating various diseases 21. We, therefore, examined the polarization of the residual BM-Mφ population post-irradiation. As shown in Figure 1F and Figure 1G, on day 3 post-irradiation, the percentage of M1-type-like (CD11c^+^CD206^-^) BM-Mφs was slightly increased, from 2.7% to 4.0% (*P* < 0.05), while the percentages of M1/M2-type (CD11c^+^CD206^+^) BM-Mφs and M2 (CD11c^-^CD206^+^) BM-Mφs were greatly increased, approximately five-fold (from 1.7% to 9.7%, *P* < 0.001) and approximately three-fold (from 5.5% to 16.3%, *P* < 0.05), respectively. This dataset suggests that the residual BM-Mφs were activated, with M1/M2- and M2-type-like polarization.

### Depletion of residual BM-Mφs impeded hematopoietic regeneration post-irradiation

**Figure 2:**
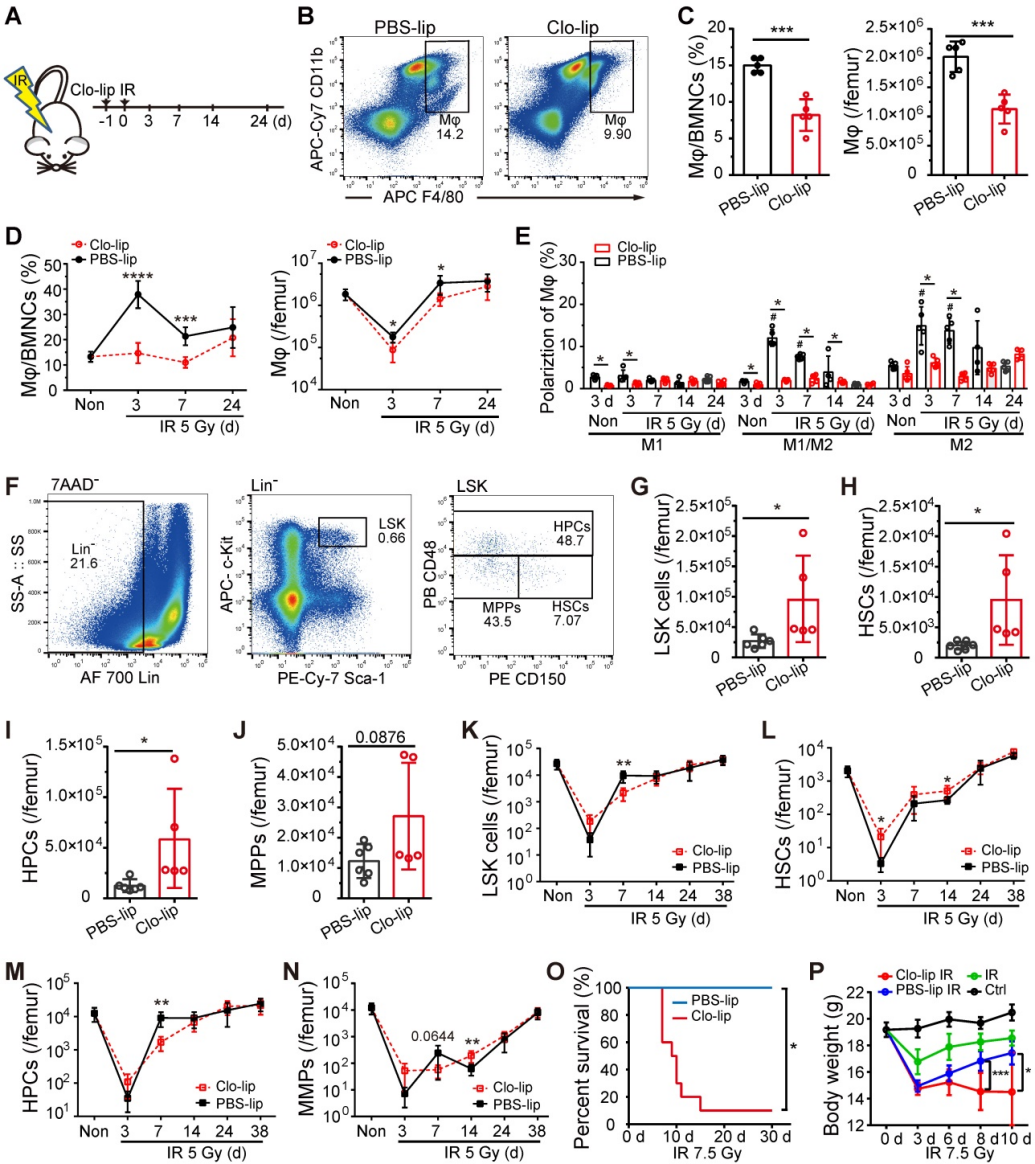
**Depletion of residual BM-Mφs impedes hematopoietic regeneration. (A)** Schematic of experimental design for Mφs depletion and irradiation treatment. **(B)** Representative flow cytometry analysis of BM-Mφs (CD11b^+^F4/80^+^) in mice treated with PBS- or clodronate-containing liposomes (PBS-lip or Clo-lip). **(C)** Percentage of BM-Mφs among BMNCs (left panel) and number of BM-Mφs per femur (right panel) at 3 days after PBS-lip or Clo-lip injection without irradiation (n = 5 mice, t-test).** (D)** Percentage of BM-Mφs among BMNCs (left panel) and number of BM-Mφs per femur (right panel) at different time points after 5-Gy irradiation, in mice pretreated with Clo-lip or PBS-lip (n = 6-13 mice, t-test). **(E)** Percentages of polarized BM-Mφs among all BM-Mφs. (n = 5 mice). *P < 0.05, t-test; #* P* < 0.05, Tukey test, compared to non-irradiation (Non) group with PBS-lip injection. **(F)** Representative flow cytometry analysis of hematopoietic stem/progenitor cells. **(G-J)** Number of LSKs (Lin^-^Sca-1^+^c-Kit^+^) **(G)**, HSCs (LSK CD48^-^CD150^+^) **(H)** HPCs (LSK CD48^+^CD150^+/-^) **(I),** and MPPs (LSK CD48^-^CD150^-^) **(J)** per femur at 3 days after PBS-lip or Clo-lip injection without irradiation (n = 5-6 mice, t-test). **(K-N)** Number of LSKs **(K)**, HSCs **(L)**, HPCs **(M)**, and MPPs **(N)** per femur after 5-Gy irradiation, for mice injected with Clo-lip vs. PBS-lip (n = 4-7 mice, t-test). **(O-P)** Kaplan-Meier survival curve (n = 10 mice, Log-rank test) **(O)** and body weight **(P)** for mice with Mφs depletion and 7.5-Gy irradiation treatment. Graphs show mean ± SD. *P < 0.05; **P < 0.01; ***P < 0.001.

To deplete BM-Mφs, we injected mice with 200 μL Clo-lip via tail vein one-day pre-irradiation (Figure 2A). As shown in Figure 2B and C, BM-Mφ levels were significantly reduced in the Clo-lip injection group compared with the PBS-lip injection group on the third day under non-irradiation conditions. Under the irradiation condition, Clo-lip injection also reduced the count and proportion of residual BM-Mφs in the first week (Figure 2D). Due to the single injection of Clo-lip and the capacity for recovery of hematopoiesis after moderate hematopoietic injury, BM-Mφs in both the Clo-lip injection group and PBS-lip injection group gradually recovered after 7 days (Figure 2D). The percentage of M1-like Mφs remained at a low level throughout the entire post-irradiation period (3-24 days), while percentages of M1/M2- and M2-like BM-Mφs remained at a high level for two weeks, then fell to the level observed in non-irradiated mice (Figure 2E). Notably, Clo-lip injection suppressed the observed increase in the percentages of M1/M2- and M2-like BM-Mφs (Figure 2E). Although the percentages of activated BM-Mφs (M1-, M1/M2-, and M2-like) were not high under non-irradiation conditions, Clo-lip injection still reduced the percentages of activated BM-Mφs (Figure 2E). Clo-lip injection thus reduced the population of BM-Mφs as well as the activation of residual BM-Mφs.

We then examined the effect of Mφ depletion on the number of hematopoietic stem/progenitor cells (HSPCs) in the bone marrow under irradiation or non-irradiation conditions with a single Clo-lip injection. Under the non-irradiation condition, we observed significant increases in bone marrow LSKs (Lin^-^Sca-1^+^cKit^+^), HSCs (LSK CD48^-^CD150^+^), and hematopoietic progenitor cells (HPCs) (LSK CD48^+^CD150^+/-^) (Figure 2F-J) in the Clo-lip injection group, compared to the PBS-lip injection group. This result suggests that BM-Mφs acted as a negative regulator of the HSC pool to maintain it at a steady-state, consistent with previous findings 9. On the third day after irradiation, residual LSK and HSC populations were greater in the Clo-lip injection group than in the PBS-lip injection group. In contrast, on day 7, the Clo-lip injection group had fewer LSKs and HPCs than the PBS-lip injection group (Figure 2K-N). These results indicate that Mφ depletion delayed HSPC pool recovery after irradiation injury. Notably, Mφ depletion was associated with a greater number of residual HSPCs on day 3 after irradiation, possibly because the damaged HSPCs had not yet been eliminated by phagocytes. After 24 days, HSPC populations in both the Clo-lip injection and PBS-lip injection groups gradually recovered, with no significant difference between groups (Figure 2K-N), further supporting the recovery of bone marrow BM-Mφs in both groups. Considering that a single dose of Clo-lip for Mφ depletion did not completely remove the capacity for hematopoietic regeneration after irradiation with 5 Gy, we assessed the effect of a single dose of Clo-lip for Mφs depletion under 7.5-Gy irradiation. As shown in Figure 2O, in the Clo-lip injection group, 9 out of 10 mice died within 7-15 days, whereas no mice died in the PBS-lip injection group within 30 days. In addition, bodyweight reduction was observed in all irradiation-exposed mice within three days, but only mice in the Clo-lip injection group did not tend to regain weight (Figure 2P). The flow cytometry results showed less recovery of LSK cells in Clo-lip-injected mice than in PBS-lip-injected mice on day 7 after irradiation exposure (Figure S1). These results indicate that residual BM-Mφs played a positive role in hematopoietic regeneration after irradiation.

### Residual BM-Mφs play a critical role in sinusoidal regeneration by secreting VEGF-A after irradiation

**Figure 3:**
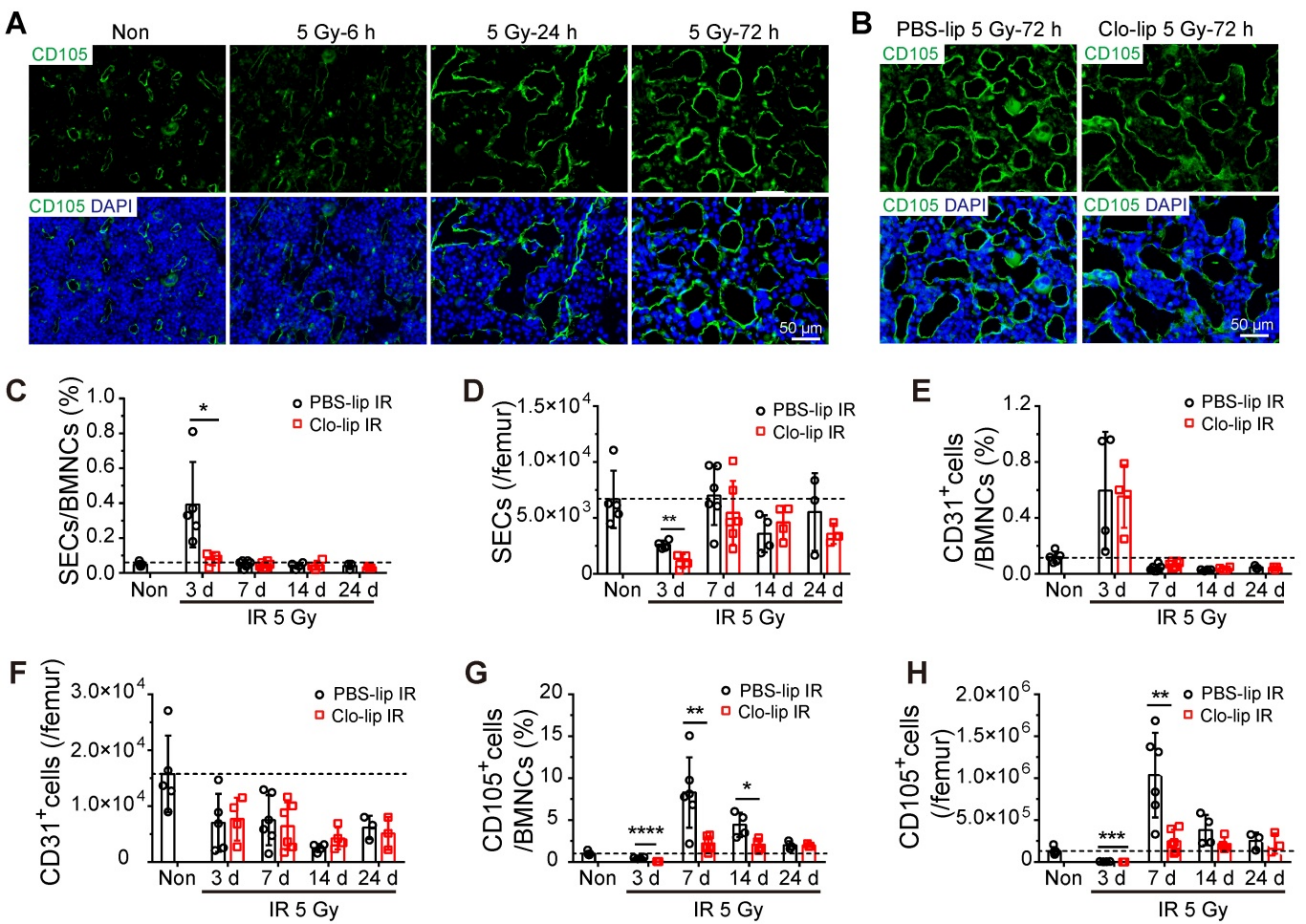
** Depletion of residual BM-Mφs impedes sinusoidal endothelial cell (SEC) regeneration. (A)** *In situ* immunofluorescence showing bone marrow sinusoids (green, CD105) after 5-Gy irradiation. Nuclei were stained with DAPI (blue). **(B)** Immunofluorescence images of bone marrow sinusoids (green, CD105) at 3 days after 5-Gy irradiation in PBS controls and mice with Mφs depletion by Clo-lip. Scale bar, 50 μm. **(C-H)** Flow cytometry analysis to determine relative percentage of SECs (CD45^-^Ter119^-^CD31^+^CD105^+^) among BMNCs **(C)**, number of SECs per femur **(D)**, relative percentage of CD31^+^ ECs (CD45^-^Ter119^-^CD31^+^CD105^-^) among BMNCs **(E)**, number of CD31^+^ ECs per femur **(F)**, relative percentage of CD105^+^ stromal cells (CD45^-^Ter119^-^CD31^-^CD105^+^) among BMNCs **(G)** and number of CD105^+^ stromal cells per femur **(H)** at indicated times after 5-Gy irradiation and pretreatment with PBS- or Clo-lip injection (n = 3-6 mice). Data are shown as mean ± SD. *P < 0.05; **P < 0.01; ***P < 0.001 (t-test).

To explore the mechanism by which BM-Mφs support hematopoietic recovery post-irradiation, we assessed the effect of Mφ depletion on sinusoidal regeneration. Immunofluorescence staining showed that bone marrow sinusoidal dilation occurred as early as 6 h post-irradiation, and obvious sinusoidal dilation and rupture could be seen 24 h post-irradiation (Figure 3A). On the third day, although sinusoidal dilation still existed, there was obvious sinusoidal recovery, with the emergence of complete vascular morphology (Figure 3A). However, at three days post-irradiation, the Clo-lip injection group had significantly more severe sinusoidal dilation and rupture than the PBS-lip group, suggesting that the structural recovery of sinusoids was hindered by BM-Mφ depletion (Figure 3B). Flow cytometry analysis of sinusoidal endothelial cells (SECs) in bone marrow, identified as Ter119^-^CD45^-^CD31^+^CD105^+^ cells, confirmed that sinusoidal vessel recovery was delayed (Figure S2A). The relative percentage and number of SECs in the Clo-lip injection group were remarkably decreased, compared with the PBS-lip injection group, on the third day after irradiation (0.39 ± 0.11% vs. 0.08 ± 0.02%, *P* < 0.05; 2598 ± 187/femur vs. 1131 ± 312/femur, *P* < 0.01) (Figure 3C-D). After 7 days, SECs in both groups gradually recovered to the levels observed in non-irradiated mice (Figure 3C-D). In contrast, there was no difference in the proportion or number of CD31^+^ vascular cells (Ter119^-^CD45^-^CD31^+^CD105^-^) between the Clo-lip injection and PBS-lip injection groups at all-time points after irradiation (Figure 3E-F). Moreover, CD31^+^ vascular cells in both groups failed to recover to non-irradiation levels. Interestingly, the recovery of CD105^+^ stromal cells (Ter119^-^CD45^-^CD31^-^CD105^+^) after irradiation was delayed after BM-Mφ depletion, similar to the delay observed for SECs (Figure 3G-H).

Notably, ECs are known to display phagocytic ability in association with the macrophage recruitment that occurs during fibrosis after neural injury 22 and use a formin-dependent phagocytosis-like process to internalize the bacterium *Listeria monocytogenes* 23. To verify the effect of SEC phagocytosis of Clo-lip in delaying sinusoidal regeneration, we injected DiI-labeling liposomes (DiI-lip) into mice via tail vein. After 24 h, we observed no uptake of DiI-lip in CD105^+^ SECs, and almost all cells that engulfed DiI-lip were F4/80^+^ BM-Mφs (Figure S2B). While flow cytometry analysis showed that under non-irradiation conditions, more than half of BM-Mφs were depleted one day after Clo-lip injection (Figure S2C), Clo-lip injection did not significantly alter the sinusoid structure or the number of SECs (Figure S2D-E). This result is consistent with previous research showing that BM-Mφs are the predominant phagocytic population in bone marrow 24. These results indicate that the delay in sinusoidal regeneration after irradiation associated with Clo-lip injection was caused by the depletion of BM-Mφs rather than the direct depletion of SECs.

**Figure 4:**
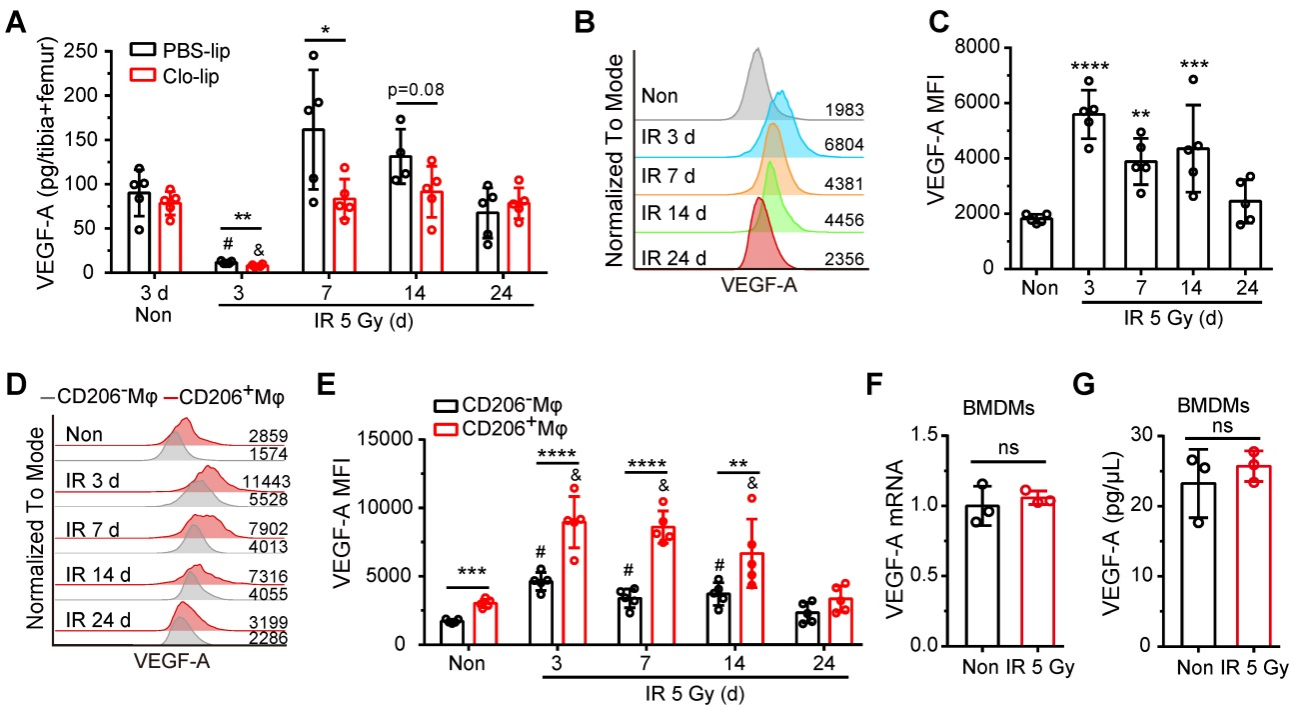
** Residual BM-Mφs upregulate VEGF-A after irradiation. (A)** Quantification of bone marrow VEGF-A levels by ELISA at indicated times after 5-Gy irradiation and treatment with PBS-lip or Mφs depletion by Clo-lip (n = 4-5 mice). *P < 0.05; **P < 0.01*, t*-test; # *P* < 0.05, compared to non-irradiation with PBS-lip injection, Tukey test; & *P* < 0.05, compared to non-irradiation with Clo-lip injection, Tukey test. **(B)** Representative flow cytometry analysis of VEGF-A expression in BM**-**Mφs from mice after 5-Gy irradiation or no irradiation. **(C)** Histogram showing mean fluorescence intensity (MFI) of VEGF-A in BM**-**Mφs (n = 5 mice, Tukey test). **(D)** Representative flow cytometry analysis of VEGF-A expression in CD206^+^ and CD206^-^ BM-Mφs from mice after irradiation or non-irradiation, with or without Mφs depletion by Clo-lip. **(E)** Histogram showing MFI of VEGF-A in CD206^+^ and CD206^-^ BM-Mφs (n = 5 mice). **P < 0.01; ***P < 0.001*,* ***P < 0.0001,* t*-test; # *P* < 0.05, compared to non-irradiation with PBS-lip injection, Tukey test; & *P* < 0.05, compared to non-irradiation with Clo-lip injection, Tukey test. Data are from one experiment and representative of three independent experiments. **(F)** RT-PCR analysis of VEGF-A mRNA levels in BMDMs 24 h after irradiation or non-irradiation *in vitro* (n = 3 independent experiments, *t*-test). **(G)** Quantification by ELISA of VEGF-A expression in BMDMs 24 h after irradiation or non-irradiation (n = 3 independent experiments, *t*-test). Data are shown as mean ± SD; ns, not significant.

It was previously reported that the regeneration of SECs post-irradiation is dependent on activation of the VEGF-A/VEGFR2 pathway 15. We, therefore, investigated whether residual BM-Mφs serve as a vital source of VEGF-A. ELISA assays showed that VEGF-A levels in bone marrow drastically decreased within three days post-irradiation and recovered to normal levels at 7 days (Figure 4A). The deletion of BM-Mφs by Clo-lip did not affect VEGF-A content in bone marrow under non-irradiation conditions but did significantly reduce VEGF-A content in the first-week post-irradiation injury (Figure 4A). Flow cytometry analysis revealed that VEGF-A was robustly upregulated in residual BM-Mφs on day 3 after irradiation and gradually returned to normal levels by 24 days (Figure 4B-C). Notably, the expression of VEGF-A was higher in CD206^+^ BM-Mφs (including M1/M2-like and M2-like phenotype BM-Mφs) than in CD206^-^ BM-Mφs, under both non-irradiation and irradiation conditions (Figure 4D-E). In addition, VEGF-A expression was significantly upregulated after irradiation in both CD206^+^ BM-Mφs and CD206^-^ BM-Mφs after irradiation, with a gradual return to normal levels in both groups (Figure 4D-E). Interestingly, irradiation treatment could not directly induce the upregulation of VEGF-A mRNA and protein levels in BMDMs *in vitro* (Figure 4F-G), indicating that the upregulation of VEGF-A in residual BM-Mφs after irradiation *in vivo* may be related to the irradiated bone marrow microenvironment, not to a direct effect of irradiation. The above results suggest that residual BM-Mφs are essential for the regeneration of SECs, probably via the upregulation of VEGF-A expression post-irradiation.

### Mechanosensation by Piezo1 mediates upregulation of VEGF-A in residual BM-Mφs after irradiation

**Figure 5:**
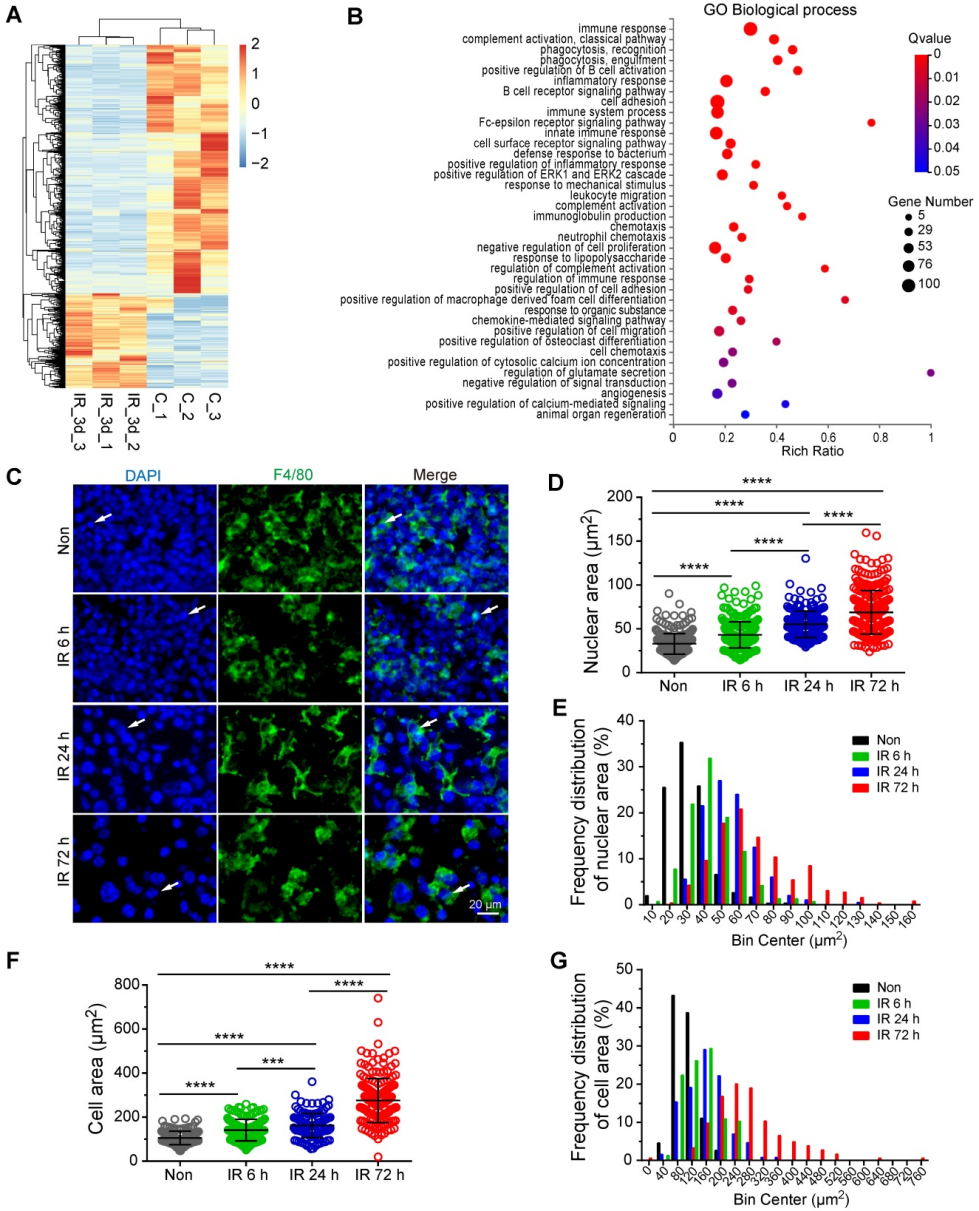
**Residual BM-Mφs respond to mechanical stretching.** BM-Mφs were sorted from mice 3 days after 5-Gy irradiation (IR_3d) or non-irradiation (C) for RNA sequencing analysis. **(A)** Heat map showing differentially expressed genes (|Log_2_(FC)| > 1, false discovery rate (FDR) < 0.05) in BM**-**Mφs from irradiation-treated mice, compared to controls. **(B)** Bubble diagram showing enriched gene ontology (GO) biological process terms for differentially expressed genes. **(C)** *In situ* immunofluorescence showing the morphology of BM-Mφs (green, F4/80) in frozen femur sections from irradiated mice. Nuclei were stained with DAPI (blue). Scale bar, 20 μm. **(D)** Nuclear area of F4/80^+^Mφs in the bone marrow. Values represent mean ± SD, n = 306, 311, 200, 260 cells, respectively, from three separate experiments. ****P < 0.0001 (Kruskal-Wallis test). **(E)** Frequency distribution of nuclear area.** (F)** Cell area of F4/80^+^Mφs in the bone marrow. Values represent mean ± SD, n = 155, 157, 131, 184 cells, respectively, from three separate experiments. ***P < 0.001; ****P < 0.0001 (Kruskal-Wallis test). **(G)** Frequency distribution of cell area.

To investigate the mechanism of VEGF-A upregulation in residual BM-Mφs post-irradiation, we sorted the BM-Mφs from mice with or without 5-Gy irradiation for transcriptome sequencing analysis. Comparison of residual BM-Mφs, three days post-irradiation with steady-state BM-Mφ populations, revealed that 687 genes were upregulated, and 1,820 genes were downregulated (| Log_2_ (FC) | > 1, FDR < 0.05) in (Figure 5A and Dataset S1). Differentially expressed genes were significantly enriched in GO biological process terms associated with the immune response, phagocytosis, cell adhesion, and chemotaxis (Figure 5B). In addition, we noticed that differentially expressed genes were also enriched in GO terms, including those genes responsive to mechanical stimulus, positive regulation of cytosolic calcium ion concentration, positive regulation of calcium-mediated signaling, and angiogenesis (Figure 5B). Considering the BMNC apoptosis caused by irradiation injury and resulting bone marrow ablation, residual BMNCs (especially BM-Mφs, the predominant residual population) may be passively stretched. As expected, immunofluorescence staining showed that the residual BM-Mφs (F4/80^+^ cells) in bone marrow were gradually stretched over time within three days post-irradiation (Figure 5C). To quantify the magnitude of the stretch of residual BM-Mφs, we analyzed the nuclear size and cell size of BM-Mφs. The average nuclear area of BM-Mφs clearly increased, from 32.83 (± 0.68) μm^2^ at steady state to 43.13 (± 0.84), 55.23 (± 1.06), and 68.72 (± 1.54) μm^2^ at 6 h, 24 h, and 72 h, respectively, post-irradiation injury (Figure 5D). The average cell area of BM-Mφs significantly increased from 105.56 (± 2.44) μm^2^ at steady state to 141.32 (± 3.89), 162.36 (± 4.79), and 275.61 (± 7.36) μm^2^ at 6 h, 24 h and 72 h, respectively, post-irradiation injury (Figure 5F). The frequency distribution of nuclear area and cell area also supports the stretching of residual BM-Mφs after irradiation (Figure 5E and G). These results suggest that residual BM-Mφs may respond to a mechanical stretching stimulus caused by changes in the physical microenvironment during irradiation injury.

**Figure 6:**
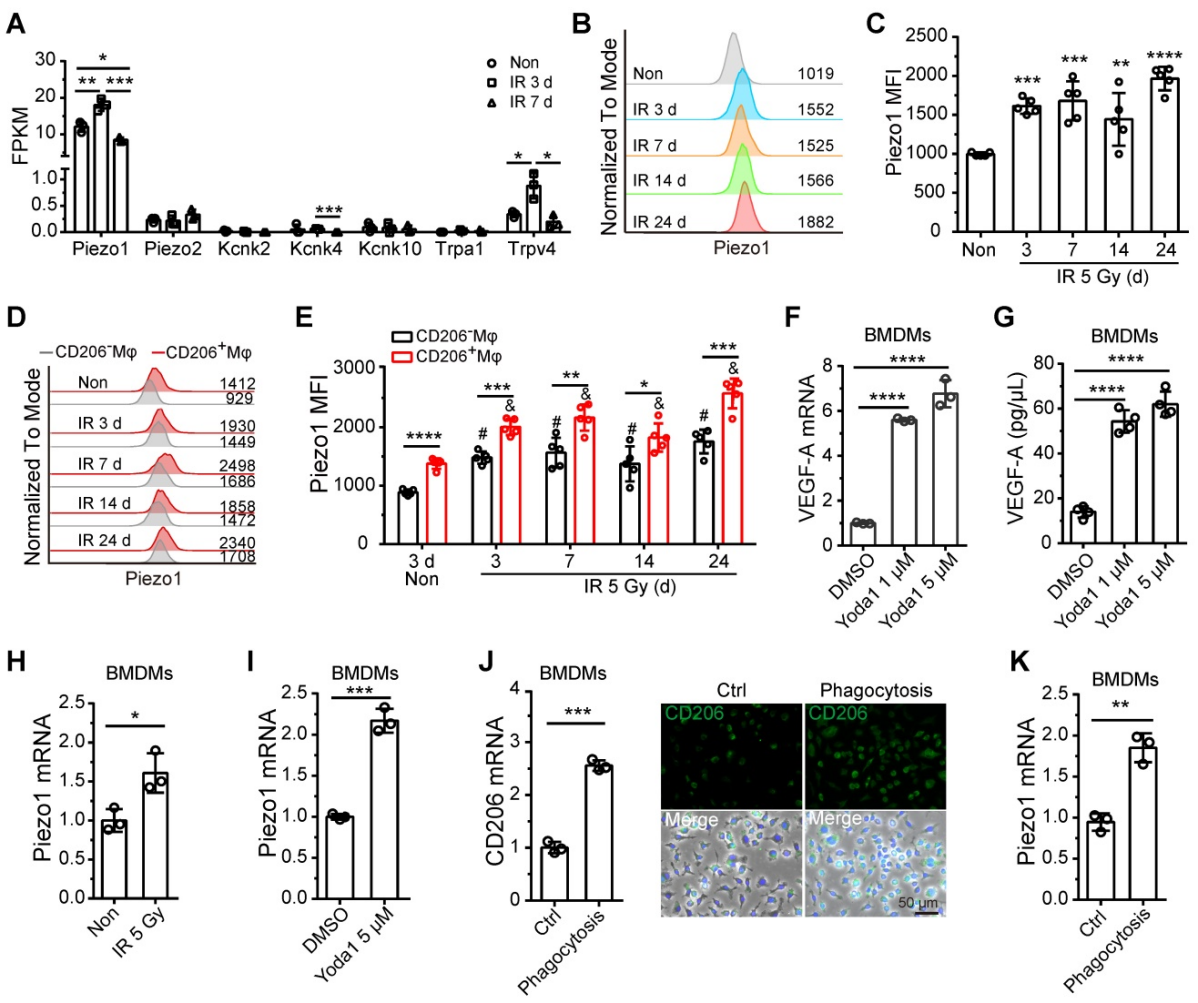
**Piezo1 activation mediates the upregulation of VEGF-A in BM-Mφs. (A)** Expression analysis of known mammalian mechanosensory ion channels in BM-Mφs after 5-Gy irradiation or non-irradiation (n = 3 mice, Tukey test). FPKM: Fragments per kilobase of transcript per million mapped fragments. **(B)** Representative flow cytometry analysis of Piezo1 expression in BM**-**Mφs after 5-Gy irradiation or non-irradiation. **(C)** Histogram showing means fluorescence intensity (MFI) of Piezo1 in BM**-**Mφs (n = 5 mice, Tukey test). **(D)** Representative flow cytometry analysis of Piezo1 expression in CD206^+^ and CD206^-^ BM-Mφs from mice after irradiation or non-irradiation with Mφs depletion by Clo-lip or not. **(E)** Histogram showing MFI of Piezo1 in CD206^+^ and CD206^-^ BM-Mφs (n = 5 mice). *P < 0.05, **P < 0.01, ***P < 0.001*,* ***P < 0.0001,* t*-test; # *P* < 0.05, compared to non-irradiated group with PBS-lip injection, Tukey test; & *P* < 0.05, compared to non-irradiated group with Clo-lip injection, Tukey test. **(F)** RT-PCR analysis of VEGF-A mRNA levels in BMDMs 6 h after Yoda1 treatment *in vitro* (n = 3 independent experiments, Tukey test). **(G)** ELISA analysis of VEGF-A expression in BMDMs 24 h after Yoda1 treatment* in vitro* (n = 3 independent experiments, Tukey test).** (H-I),** RT-PCR analysis of Piezo1 mRNA levels in BMDMs 24 h after irradiation **(H)** or Yoda1 **(I)** treatment. **(J)** RT-PCR (left panel) and immunofluorescence analysis (right panel) of CD206 expression in BMDMs 24 h after phagocytosis of irradiation-induced apoptotic bone marrow cells (n = 3 independent experiments, *t*-test). Scale bar, 50 μm. **(K)** RT-PCR analysis of Piezo1 expression in BMDMs 24 h after phagocytosis of irradiation-induced apoptotic bone marrow cells (n = 3 independent experiments, *t*-test). Data are shown as mean ± SD. *P < 0.05; **P < 0.01; ***P < 0.001; ****P < 0.0001; ns, not significant.

Recent research has demonstrated that the response of monocytes/macrophages to mechanical force is mediated by Piezo1, a mechanosensory ion channel (MSICs) with a high affinity for calcium 25. In our study, BM-Mφs had increased expression of the *Piezo1* gene and negligible levels of other MSICs, including *Piezo2*, *Kcnk2*, *Kcnk4*, *Kcnk10*, *Trpa1,* and *Trpv4* (Figure 6A and Dataset S2). Gene expression levels of *Piezo1* in BM-Mφs increased on the third day, then fell back to normal on day 7 after irradiation injury (Figure 6A). Flow cytometry analysis showed that the protein expression of Piezo1 in BM-Mφs increased at all-time points after irradiation (3-24 days) (Figure 6B-C). Notably, protein levels of Piezo1 were higher in CD206^+^ BM-Mφs than in CD206^-^ BM-Mφs under both non-irradiation and irradiation conditions (Figure D-E). In addition, the expression of Piezo1 increased after irradiation in both CD206^+^ BM-Mφs and CD206^-^ BM-Mφs (Figure D-E).

Previous reports have shown that mechanical stretching activates Piezo1 in epithelial cells 26 and human neural stem cells 27. Others have found that mechanical stretching upregulates VEGF-A in various cell types, such as osteoblastic cells 28, mesenchymal stromal cells, and periodontal ligament fibroblasts 29. Therefore, we presumed that Piezo1 activation in response to mechanical stretching might contribute to the upregulation of VEGF-A in residual BM-Mφs after irradiation. To verify this hypothesis, we used Yoda1 to treat BMDMs *in vitro*. Yoda1 is a specific chemical agonist of Piezo1 and is widely used to simulate Piezo1 activation caused by mechanical stimulation 30. Our RT-PCR and ELISA assays showed that activation of Piezo1 by Yoda1 greatly upregulated gene and protein expression of VEGF-A (Figure 6F-G). These results suggest that Piezo1 activation in BM-Mφs may contribute to the upregulation of VEGF-A after irradiation.

Further* in vitro* experiments showed that irradiation could directly upregulate Piezo1 in BMDMs (Figure 6H). The activation of Piezo1 by Yoda1 also contributed to the upregulation of Piezo1 (Figure 6I). In this study, we found that the population of CD206^+^ BM-Mφs, which includes M1/M2-like and M2-like BM-Mφs, significantly increased after irradiation, and the CD206^+^ BM-Mφs had higher Piezo1 expression than the CD206^-^ BM-Mφs. These results drove us to examine the effect of Mφ activation on Piezo1 expression. The M2-type BMDMs activated by interleukin-4 (IL-4) 21 had no significant upregulation of Piezo1 compared to the non-activated BMDMs (Figure S3A-B). M2-like activation can be induced by various factors in addition to IL-4, including the phagocytosis of apoptotic cells 31. After irradiation, a large number of bone marrow cells undergo apoptosis, which requires the phagocytosis of BM-Mφs. We found that the phagocytosis of irradiation-induced apoptotic bone marrow cells promoted an increase in the gene and protein expression of M2-type maker CD206 in BMDMs *in vitro*, suggesting that phagocytosis induced M2-like activation (Figure S4 and Figure 6J). Irradiation had no direct effect on CD206 expression but slightly upregulated the M1-type marker inducible nitric oxide synthase (iNOS) in BMDMs (Figure S5). Notably, phagocytosis-induced M2-like BMDMs had higher expression of Piezo1 than normal BMDMs (Figure 6K). These results suggest that irradiation, activation of Piezo1, and phagocytosis-induced M2-like polarizations were responsible for the upregulation of Piezo1 in residual BM-Mφs after irradiation injury. Next, we wanted to determine the molecular mechanism by which Piezo1-mediated signaling pathways regulate these processes.

### Piezo1 mediates mechanotransduction via calcineurin/NFAT/HIF-1α signaling

**Figure 7:**
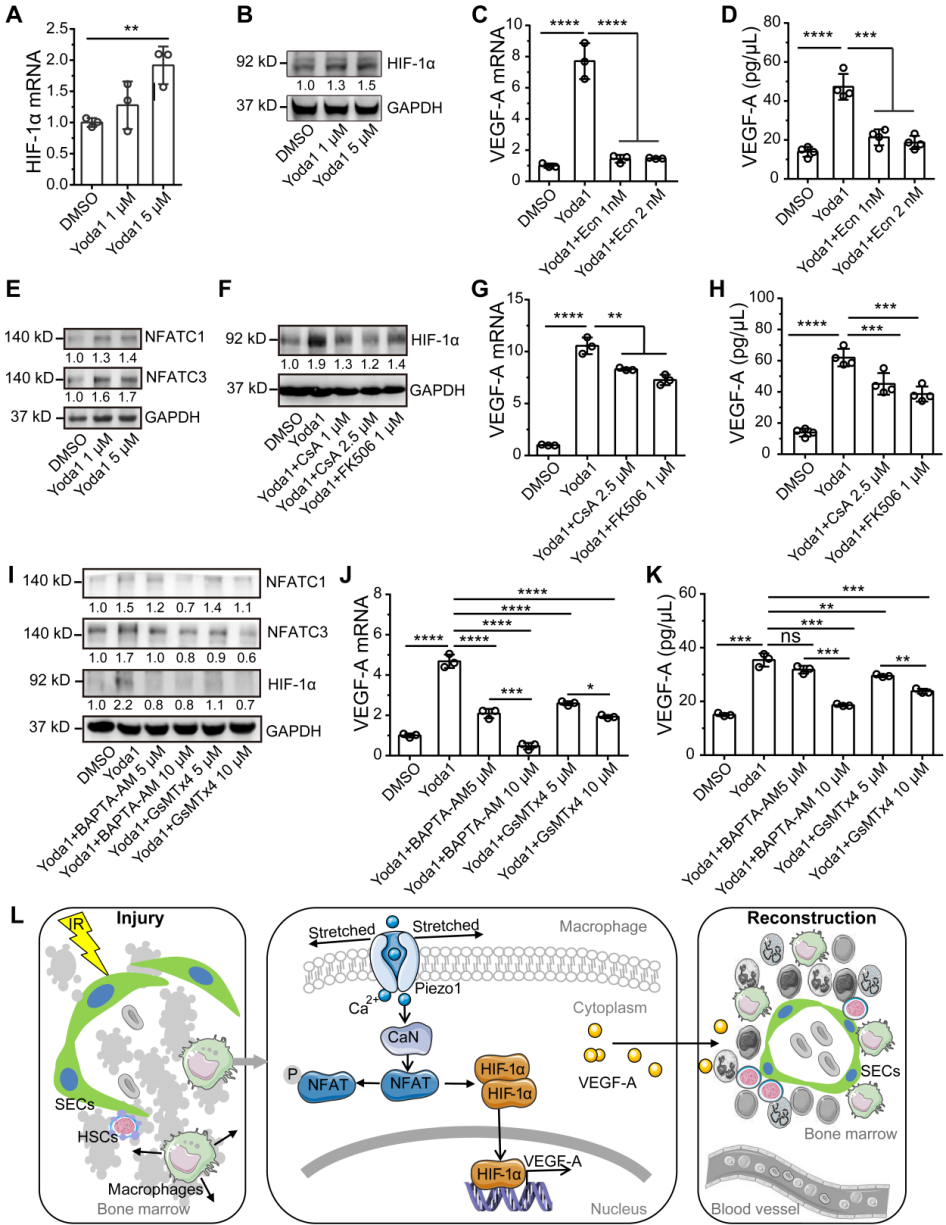
** Activation of calcineurin/NFAT/HIF-1α signaling induces a Piezo1-mediated increase in VEGF-A expression in BMDMs. (A)** RT-PCR analysis of HIF-1α mRNA levels in BMDMs 6 h after Yoda1 treatment (n = 3 independent experiments, Tukey test). **(B)** Western blot analysis of HIF-1α accumulation in BMDMs 24 h after Yoda1 treatment. Blots are representative of three independent experiments. **(C)** RT-PCR analysis of VEGF-A mRNA levels in BMDMs pretreated with echinomycin (Ecn) for 30 min before 6-h Yoda1 (5 μM) treatment (n = 3 independent experiments, Tukey test). **(D)** ELISA analysis of VEGF-A expression in BMDMs pretreated with Ecn for 30 min before 24-h Yoda1 (5 μM) treatment (n = 3 independent experiments, Tukey test). **(E)** Western blot analysis of NFATC1 and NFATC3 in BMDMs treated with Yoda1 for 24 h. **(F)** Western blot analysis of HIF-1α in BMDMs pretreated with CsA or FK506 for 30 min before 24-h Yoda1 (5 μM) treatment. Blots are representative of three independent experiments. **(G)** RT-PCR analysis of VEGF-A mRNA levels in BMDMs pretreated with CsA or FK506 for 30 min before 6-h Yoda1 (5 μM) treatment (n = 3 independent experiments, Tukey test). **(H)** ELISA analysis of VEGF-A expression in BMDMs pretreated with CsA or FK506 for 30 min before 24-h Yoda1 (5 μM) treatment (n = 3 independent experiments, Tukey test). **(I)** Western blot analysis of NFATC1 and NFATC3 in BMDMs pretreated with BAPTA-AM or GsMTx4 for 30 min before 24-h Yoda1 (5 μM) treatment. Blots are representative of three independent experiments. **(J)** RT-PCR analysis of VEGF-A mRNA levels in BMDMs pretreated with BAPTA-AM or GsMTx4 for 30 min before 6-h Yoda1 (5 μM) treatment (n = 3 independent experiments, Tukey test). **(K)** ELISA analysis of VEGF-A expression in BMDMs pretreated with BAPTA-AM or GsMTx4 for 30 min before 24-h Yoda1 (5 μM) treatment (n = 3 independent experiments, Tukey test). Data are shown as mean ± SD. *P < 0.05; **P < 0.01; ***P < 0.001; ****P < 0.0001. **(L)** Schematic model showing that Piezo1-mediated mechanosensation in BM-Mφs promotes vascular niche regeneration after irradiation injury.

Hypoxia-inducible factor 1α (HIF-1α) is a potent transcriptional regulator of VEGF-A and can be regulated by mechanical stresses in myocardial tissue and as part of the innate immune response 25, 32. So, we examined the role of HIF-1α in Piezo1 activation-mediated upregulation of VEGF-A. Piezo1 activation by Yoda1 slightly upregulated HIF-1α gene expression and significantly promoted the accumulation of HIF-1α protein in BMDMs (Figure 7A-B, S6A). Pretreatment with Ecn, an inhibitor of HIF-1α-DNA interactions 33, resulted in a loss of Yoda1-induced VEGF-A expression at the mRNA and protein levels (Figure 7C-D). These results demonstrate that Piezo1 activation upregulated VEGF-A via HIF-1α accumulation.

We next sought to determine how Piezo1 activation induced the accumulation of HIF-1α. We first investigated whether AKT or ERK1/2 signaling was involved since both molecules are known to respond to Piezo1-mediated calcium influx 26 and participate in HIF-1α-regulated mechanotransduction via PI3K 32 or VEGF 34. We found that Yoda1 increased the phosphorylation of AKT and ERK1/2; SCH772984, an inhibitor of ERK1/2, reduced phosphorylation of ERK1/2 and AKT but had no effect on Yoda1-induced HIF-1α accumulation (Figure S7A), suggesting that Piezo1 activation-mediated HIF-1α accumulation was not mediated by AKT or ERK1/2 signaling. Next, we examined the role of calcineurin/nuclear factor of activated T cells (NFAT) signaling in Yoda1-induced HIF-1α accumulation and VEGF-A upregulation because activation of the calcineurin/NFAT signaling pathway depends on elevated intracellular calcium, and Piezo1 activation can induce this process through concerted activation of NFAT-YAP1-ß-catenin 35. BM-Mφs had high *Nfatc1* and *Nfatc3* gene expression and negligible *Nfatc2*, *Nfatc4,* and *Nfat5* gene expression (Figure S7B and Dataset S2). Yoda1 induced the accumulation of NFATC1 and NFATC3 proteins in BMDMs *in vitro* (Figure 7E, S6B-C). The inhibition of calcineurin/NFAT signaling by CsA and FK506 caused the loss of Yoda1-induced HIF-1α accumulation in BMDMs (Figure 7F, S6D) and decreased Yoda1-induced VEGF-A expression, at both the gene and protein levels (Figure 7G-H). Similarly, in RAW264.7 cells, pretreatment with CsA may inhibit Yoda1-induced accumulation of HIF-1α, NFATC1, and NFATC3 proteins (Figure S8A); both CsA and Ecn suppressed Yoda1-induced VEGF-A expression (Figure S8B-C).

The activation of Piezo1 by Yoda1 (5 μM) can induce rapid calcium influx in BMDMs, leading to CaMKII-Mst1/2-Rac-regulated pathogen ingestion 36. To verify whether Piezo1-mediated calcium influx is involved in activating NFAT/HIF-1α/VEGF-A signaling induced by Yoda1 in BMDMs, we used GsMTx4, an inhibitor of Piezo1, and BAPTA-AM, a cell-permeable calcium chelator. Pretreatment of GsMTx4 and BAPTA-AM suppressed the Yoda1-induced accumulation of HIF-1α, NFATC1, and NFATC3 proteins (Figure 7I, S6E-G) and also suppressed Yoda1-induced VEGF-A expression at the mRNA and protein levels (Figure 7J-K). These results suggest that Piezo1 activation upregulated VEGF-A via calcineurin/NFAT/HIF-1α signaling.

## Discussion

Our study examined the role and signaling mechanism of BM-Mφs in hematopoietic reconstruction and sinusoidal vascular regeneration after irradiation. The results suggest that irradiation induces membrane stretch of residual BM-Mφs that are sensed by the mechanosensitive Piezo1 channel that mediates calcium entry and subsequent stimulation of the calcineurin/NFAT/HIF-1α signaling pathway, leading to expression and release of VEGF-A. The upregulated VEGF-A expression in BM-Mφs promotes sinusoidal regeneration. The findings are novel and insightful, as our study first demonstrated that residual BM-Mφs are essential to vascular niche recovery and hematopoietic reconstitution post-irradiation injury as BM-Mφs can recognize and respond to the physical environment (Figure 7L). In addition, our study establishes the critical position of BM-Mφs in vascular niches of HSCs, with possible implications for endosteal niches 5 and MSC niches 6.

Mφs are distributed widely in many tissue types and function in homeostasis. The heterogeneity and versatility of Mφs allow this unique cell population to assume different roles under physiological vs. disease conditions. Under normal physiological conditions, Mφs mainly play a scavenging role in the phagocytosis of senescent cells 37, but under pathological conditions, they also perform diverse functions such as antigen presentation, inflammation initiation, and regression, and tissue homeostasis 38. Previous studies have shown that, during hematopoietic development, Mφs play a positive role in HSC generation, expansion, and maturation in the aorta-gonad-mesonephros and the embryonic head 39 and support the activity of pro-inflammatory aorta-associated macrophages 40. In the steady-state bone marrow HSC niche, Mφs maintain the retention and quiescence of HSCs, reducing the likelihood of excessive proliferation and differentiation, or even exhaustion, of HSCs. Under some disease states, such as infection and severe aplastic anemia, an IFN-γ-dependent increase in BM-Mφs mediates the loss of HSCs 9. In our model of bone marrow injury, the BM-Mφ population decreased due to irradiation-induced apoptosis. This reduction in the BM-Mφ population benefits the recovery of the HSC pool by releasing the inhibitory effect on HSC proliferation. Moreover, residual BM-Mφs upregulated VEGF-A, promoting regeneration of the HSC vascular niche upon irradiation injury. The results from these lines of research indicate that BM-Mφs play pleiotropic roles in the HSC niche to maintain hematopoietic homeostasis.

In this study, we found that the depletion of BM-Mφs caused a significant reduction in VEGF-A levels in bone marrow following irradiation, but not under non-irradiated, steady-state conditions. This result indicates that BM-Mφs are a key contributor to VEGF-A dynamics in bone marrow post-irradiation. In contrast, under steady-state conditions, the contribution of BM-Mφs to VEGF-A levels in bone marrow was negligible. These findings suggest that the bone marrow cell population comprises mainly BM-Mφs during the early stage of irradiation injury but not under normal conditions. The upregulation of VEGF-A in BM-Mφs after irradiation injury is mainly due to the increased population of CD206^+^ BM-Mφs (M1/M2-like and M2-like), which express higher levels of VEGF-A than do CD206^-^ BM-Mφs. The M1-type and M2-type represent two extreme poles of a broad spectrum of Mφs activation states 21 and are involved in the outcome of many diseases 41. The increase in the population of CD206^+^ BM-Mφs after irradiation in our study agrees with the previous observation that there is an increase in Arg1 activity in irradiated BM-Mφs, compared with BM-Mφs from non-irradiated C57BL/6 mice 42, as both CD206 and Arg1 are markers of M2-like activation 41. Many factors can induce the M2 activation of Mφs, including Th2-type cytokines (IL-4, IL-10, IL-13), transforming growth factor-β (TGF-β), apoptotic cells, pathogenic fungi, and nematodes 20. Our study suggests that M2-like activation of residual BM-Mφs after irradiation injury is due to the phagocytosis of apoptotic bone marrow cells, not due to a direct effect of irradiation. *In vitro* study has shown that M2-like activation may promote HSC self-renewal and expansion, in part, through the Arg1-mediated production of spermidine 43. These findings suggest that M2-like activation benefits hematopoietic reconstitution upon post-irradiation injury. It will be interesting to determine whether M2-like activation upon post-irradiation injury has other effects on hematopoietic reconstitution.

Previous reports have shown that the depletion of BM-Mφs results in a loss of endosteal osteoblasts 5, which are essential for maintaining HSCs in bone marrow 44, and in this study, we found that depletion of BM-Mφs can impede the regeneration of CD105^+^ SECs. CD105^+^ SECs are known to promote angiogenesis, osteogenesis, and the expansion of HSCs through the secretion of IL-33 45. In contrast, CD31^+^ vascular cells are not affected by Mφs depletion. Moreover, the CD31^+^ vascular cell population does not recover to baseline upon post-irradiation injury, regardless of whether the BM-Mφs have been depleted. Bone marrow Nestin^+^ MSCs (Ter119^-^CD45^-^CD31^-^Nestin^+^) are essential constituents of HSC niches 46 that promote the retention and self-renewal of HSCs by expressing niche-supporting factors such as CXCL12 and stem cells factor (SCF) via PDGFRα-CD51-regulated pathways 47. The results from this study also showed that loss of BM-Mφs after Clo-lip injection also caused a significant loss of CD105^+^ stromal cells in the bone marrow, both at a steady state and after irradiation injury. CD105 is a relatively specific marker for MSCs; 65% of nestin^+^ MSCs express CD105 48. It is unclear whether these CD105^+^ stromal cells and hematopoietic-supporting MSCs are the same cell subpopulation. The mechanism of the loss of CD105^+^ stromal cells mediated by Mφs depletion and related effects on the hematopoietic niche must be further determined.

In this study, irradiation-induced VEGF-A expression in BM-Mφs *in vivo* but not in BMDMs *in vitro*, suggesting that the mechanism of VEGF-A upregulation in BM-Mφs after irradiation was related to the pathological microenvironment in the bone marrow. Although mechanical cues (e.g., shear stress, pressure, stretch, matrix stiffness, and topography) and biochemical signals affect tissue and cell function under physiological and pathological microenvironments, our RNA-sequencing results suggested altered function related to mechanical stimulus and calcium-mediated signaling in BM-Mφs after irradiation. Numerous studies have shown that the physical environment regulates Mφ phenotype 49, Mφ phagocytosis 50, Mφ shape 51, Mφ immunoregulation 25, and Mφ migration on stiff materials 52. Mechanical stimulation needs to be converted into biochemical signals for biological effect, and MSICs and intracellular calcium are essential mediators in the process of mechanotransduction 53. Piezo1 is an MSIC with a high affinity for calcium. A variety of mechanical stimuli can activate Piezo1 and induce calcium influx, including cyclical pressure 53, matrix stiffness 54, 55, mechanical stretching 26, 27, and shear stress 56. In this study, Piezo1 was highly expressed in BM-Mφs and was upregulated after irradiation injury. However, the upregulation of Piezo1 was not sufficient to upregulate VEGF-A in the absence of mechanical stimulation. We observed no change in the cell morphology of BMDMs after irradiation* in vitro* (Figure S9), which explains why irradiation upregulated Piezo1 but failed to upregulate VEGF-A in BMDMs *in vitro*. In the early stage of bone marrow damage and repair after irradiation, the BMNC population had been ablated, and regenerative cells had not yet filled the bone marrow cavity. The remaining cells were passively stretched, resulting in the activation of Piezo1. Then, when the bone marrow cavity was filled by regenerative cells, there was a lack of mechanical stimulation, and VEGF-A expression in BM-Mφs returned to normal levels. However, Piezo1 expression in BM-Mφs was still higher in irradiated vs. non-irradiated mice. These findings demonstrate that Piezo1 activation was mediated by stretching in BM-Mφs upon bone marrow ablation after irradiation injury.

Piezo1 activation by a chemical agonist, Yoda1, increased VEGF-A expression in BMDMs* in vitro*. This effect was mediated by the accumulation of HIF-1α. HIF-1α accumulation can be induced by hypoxia via the inhibition of HIF-1α ubiquitin degradation 57. Bone marrow is a relatively hypoxic environment at a steady state. Unexpectedly, irradiation or chemotherapy does not aggravate bone marrow hypoxia, resulting in a substantial elevation in the overall bone marrow oxygen concentration 58. This phenomenon suggests that the upregulation of VEGF-A in BM-Mφs upon irradiation injury is unrelated to hypoxia.

TRPV4 can be activated by mechanical stretch and causes calcium influx 59. Recent studies have suggested that Piezo1 activation triggers TRPV4 channel opening, which is responsible for the sustained elevation in intracellular calcium in pancreatic acinar cells 60 and ECs 61 due to shear stress. Our RNA-sequencing results showed that TRPV4 was significantly upregulated in BM-Mφs after irradiation, although the expression of TRPV4 in BM-Mφs is extremely low relative to that of Piezo1 (Figure 6A). Therefore, we examined the role of TRPV4 in Yoda1-mediated VEGF-A expression in BMDMs. Notably, Piezo1 is highly expressed in BMDMs, whereas TRPV4 expression is negligible 25. In this study, the activation of TRPV4 by its agonist GSK1016790A failed to induce VEGF-A expression in BMDMs (Figure S10A-B). In addition, pretreatment with RN-1734, an inhibitor of TRPV4, could not suppress Yoda1-induced VEGF-A expression (Figure S10C-D). These results suggest that TRPV4 was not involved in Piezo1-induced VEGF-A expression.

We found that calcium influx-mediated calcineurin/NFAT signaling is involved in HIF-1α accumulation, which is in line with previous findings that NFATs, as transcription factors, transcriptionally regulate HIF-1α expression 62. Piezo1 activation induced by a low concentration of Yoda1 did not upregulate HIF-1α mRNA expression but strongly activated NFATC1 and NFATC3, which in turn promoted HIF-1α accumulation and upregulated VEGF-A expression. The mechanism by which the activation of calcineurin/NFAT signaling mediated the accumulation of HIF-1α remains to be elucidated. A transgenic mouse model with a conditional Piezo1 knockout in Mφs will be essential to verify the role of Piezo1-mediated VEGF-A upregulation in sinusoidal regeneration and hematopoietic reconstitution after irradiation injury *in vivo*.

## Conclusion

Our study shows that irradiation injury activates residual BM-Mφs to upregulate VEGF-A via Piezo1-mediated mechanosensation to promote sinusoidal regeneration and hematopoietic reconstitution. This work provides a novel role in maintaining hematopoietic homeostasis for BM-Mφs in the HSC niche.

## Supplementary Material

Supplementary figures.

## Figures and Tables

**Table 1:** Primer sequences

Gene symbol	Sequence (5′ to 3′)	
VEGF-A	Sense:	GCACATAGAGAGAATGAGCTTCC
	Antisense:	CTCCGCTCTGAACAAGGCT
HIF-1α	Sense:	ACCTTCATCGGAAACTCCAAAG
	Antisense:	ACTGTTAGGCTCAGGTGAACT
CD206	Sense:	GAGGGAAGCGAGAGATTATGGA
	Antisense:	GCCTGATGCCAGGTTAAAGCA
iNOS	Sense:	GTTCTCAGCCCAACAATACAAGA
	Antisense:	GTGGACGGGTCGATGTCAC
GAPDH	Sense:	AGGTCGGTGTGAACGGATTTG
	Antisense:	TGTAGACCATGTAGTTGAGGTCA

## Abbreviations

BMDM: Bone marrow-derived macrophage
BM-Mφ: Bone marrow-resident macrophage
BM-Mφs: Bone marrow-resided macrophages
BMNCs: Bone marrow-nucleated cells
CsA: Cyclosporin A
DEG: Differentially expressed gene
Ecn: Echinomycin
EC: Endothelial cell
EDN1: Endothelin 1
EDTA: Ethylene diamine tetra-acetic acid
FBS: Fetal bovine serum
FDR: False discovery rate
FPKM: Fragments per kilobase of transcript per million mapped fragments
GO: Gene ontology
HIF-1α: Hypoxia inducible factor 1α
HPCs: Hematopoietic progenitor cells
HSCs: Hematopoietic stem cells
IFN-γ: Interferon gamma
IL-4: Interleukin-4
iNOS: Inducible nitric oxide synthase
M-CSF: Macrophage colony-stimulating factor
MFI: Mean fluorescence intensity
MPP: Multipotent progenitor
MSC: Mesenchymal stem cell
MSICs: Mechanosensory ion channel
Mφ: Macrophage
NFAT: Nuclear factor of activated T-cells
P/S: Penicillin/streptomycin
PFA: Paraformaldehyde
PGE2: Prostaglandin E2
ROS: Reactive oxygen species
RT: Room temperature
RT-PCR: Real-time polymerase chain reaction
SCF: Stem cell factor
SEC: Sinusoidal endothelial cell
VEGF-A: Vascular endothelial growth factor A
VEGFR2: Vascular endothelial growth factor receptor 2
