# Supplementary material for: Piezo1-mediated mechanosensation in bone marrow macrophages promotes vascular niche regeneration after irradiation injury
Source: Theranostics. 2022 Jan 16;12(4):1621–38. doi: 10.7150/thno.64963 (PMC8825582; doi:10.7150/thno.64963)
Supplement: Supplementary file 1 — Supplementary figures. [file thnov12p1621s1.pdf]

# Supplemental Materials

## Piezo1-mediated mechanosensation in bone marrow macrophages promotes vascular niche regeneration after irradiation injury

Xiaomei Zhang[1], Lijia Hou[1], Fengjie Li[1], Weiwei Zhang[1], Chun Wu[1], Lixin Xiang[1], Jiuxuan Li[1], Luping Zhou[1], Xiaojie Wang[1], Yang Xiang[1], Yanni Xiao[2], Shengwen Calvin Li[3,4], Li Chen[1*], Qian Ran[1*], Zhongjun Li[1*]

[1] Laboratory of Radiation Biology, Laboratory Medicine Center, Department of Blood Transfusion, The Second Affiliated Hospital, Army Military Medical University, Chongqing 400037, China
[2] 111 Project Laboratory of Biomechanics and Tissue Repair, College of Bioengineering, Chongqing University, Chongqing 400044, China
[3] CHOC Children's Research Institute, Children's Hospital of Orange County (CHOC); Orange, CA 92868, USA
[4] Department of Neurology, University of California-Irvine School of Medicine, Orange, CA 92868, USA

[*]**Correspondence**: iceman@tmmu.edu.cn; louise-r-q@tmmu.edu.cn; shengwel@uci.edu; zhongjunli@tmmu.edu.cn.
Laboratory of Radiation Biology, Laboratory Medicine Center, Department of Blood Transfusion, The Second Affiliated Hospital, Army Military Medical University, Xinqiao Road, Shapingba, Chongqing 400037, China

## Supplemental Figures and Legends

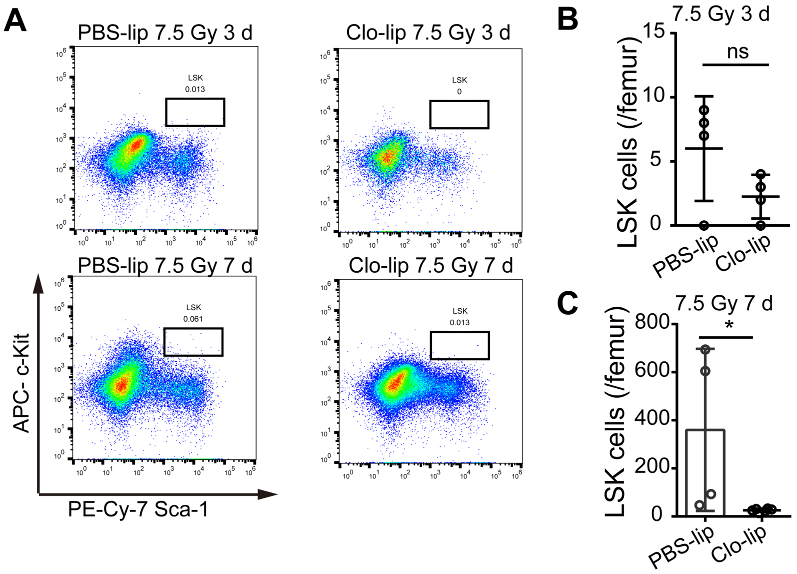


**Figure S1. Depletion of residual BM-Mφ impedes LSKs regeneration after 7.5 Gy irradiation. (A)** Representative flow cytometry analysis of LSKs in the femur after 7.5 Gy irradiation with PBS-lip or Clo-lip injection. **(B-C)** Number of LSKs per femur at 3 days **(B)** and 7 days **(C)** after 7.5 Gy irradiation with PBS-lip or Clo-lip injection (n = 4 mice, *t*-test). * $P < 0.05$; ns, not significant.

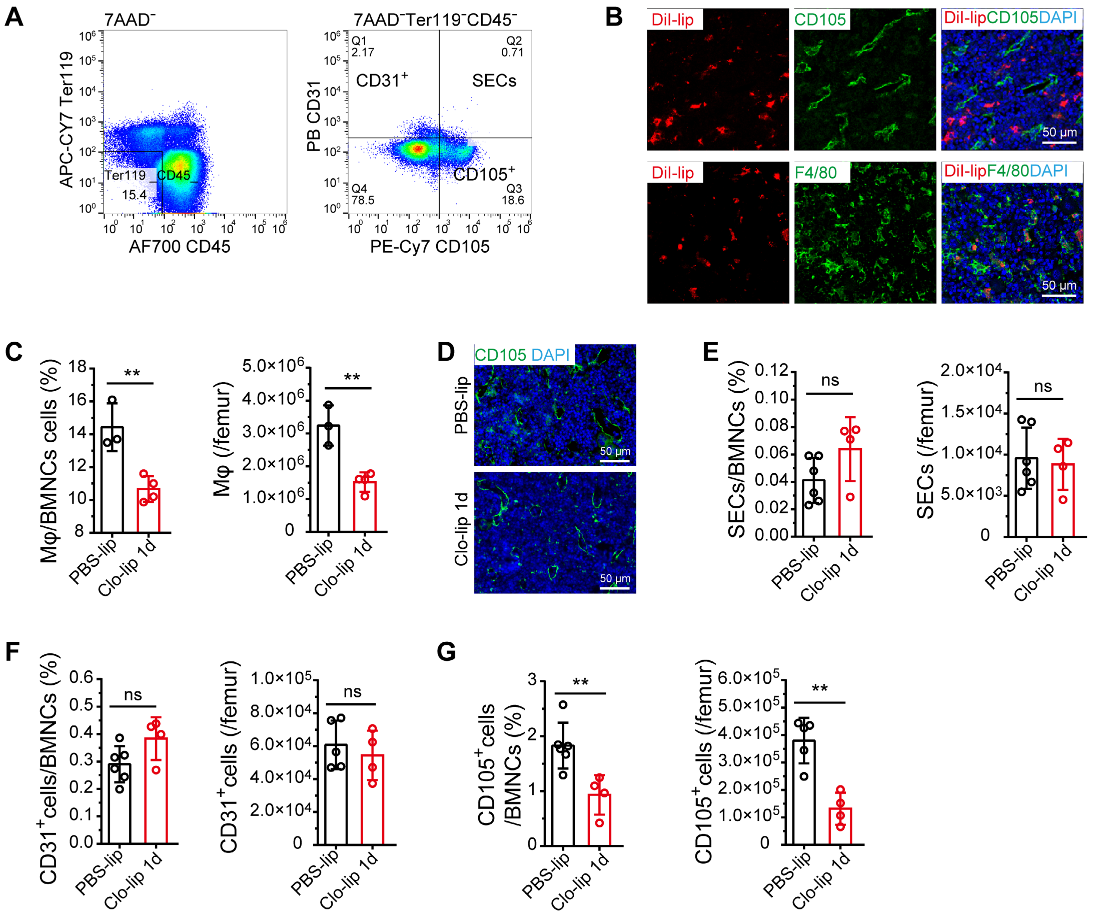


**Figure S2. Effect of BM-Mφ depletion by Clo-lip on sinusoids at steady state. (A)** Representative flow cytometry analysis of bone marrow sinusoidal endothelial cells (SECs). **(B)** *In situ* immunofluorescence images showing bone marrow cells that have engulfed DiI-labeling liposomes (DiI-lip) (red), bone marrow sinusoids (green, CD105), and BM-Mφ (green, F4/80). The nucleus was stained with DAPI (blue). Scale bar, 50 μm. **(C)** Frequency of BM-Mφ in BMNCs and number of BM-Mφ per femur. **(D)** *In situ* immunofluorescence images showing bone marrow sinusoids (green, CD105) after 1 day with Clo-lip or PBS-lip treatment under non-irradiation conditions. **(E)** Frequency of SECs in BMNCs and number of SECs per femur, **(F)** frequency of $CD31^+$ECs in BMNCs and number of $CD31^+$ECs per femur, **(G)** frequency of $CD105^+$ stromal cells in BMNCs, and the number of $CD105^+$ stromal cells per femur analyzed by flow cytometry at 1 day after Clo-lip or PBS-lip injection (n = 4–6 mice, *t*-test). Data were shown as mean ± SD. $^{**}P < 0.01$; ns, not significant.

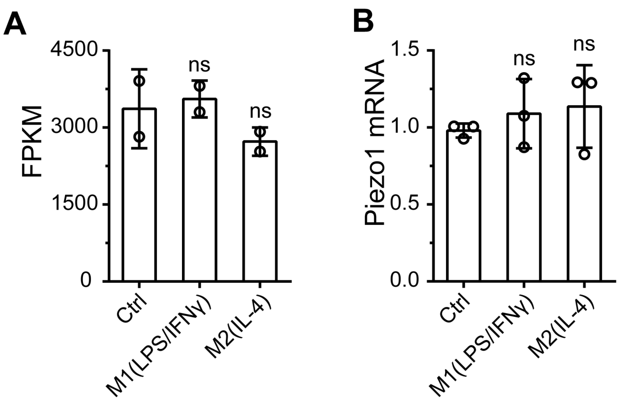


**Figure S3. Piezo1 expression in classical M1 and M2 activation in BMDMs.** BMDMs were treated with 50 ng/mL LPS and 20 ng/mL IFNγ or 100 ng/mL IL-4 for 6 h *in vitro*. **(A)** FPKM of Prizo1 in non-activated BMDMs (Ctrl), M1 and M2 BMDMs (ref: GES113836) (n = 2 independent experiments, Tukey test). **(B)** RT-PCR analysis of Piezo1 mRNA level in BMDMs after activation or not (n = 3 independent experiments, Tukey test). Data were shown as mean ± SD. ns, not significant.

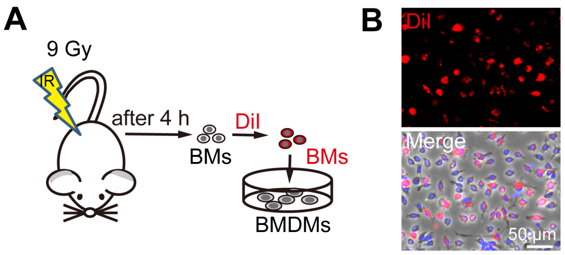


**Figure. S4 Phagocytosis of irradiation-induced apoptotic bone marrow cells (BMs) by BMDMs *in vitro*. (A)** Diagram showing phagocytosis of irradiation-induced apoptotic BMs by BMDMs *in vitro*. **(B)** Representative immunofluorescence images showing BMDMs that have engulfed DiI-labeling irradiation-induced apoptotic BMs (red). Scale bar, 50 μm.

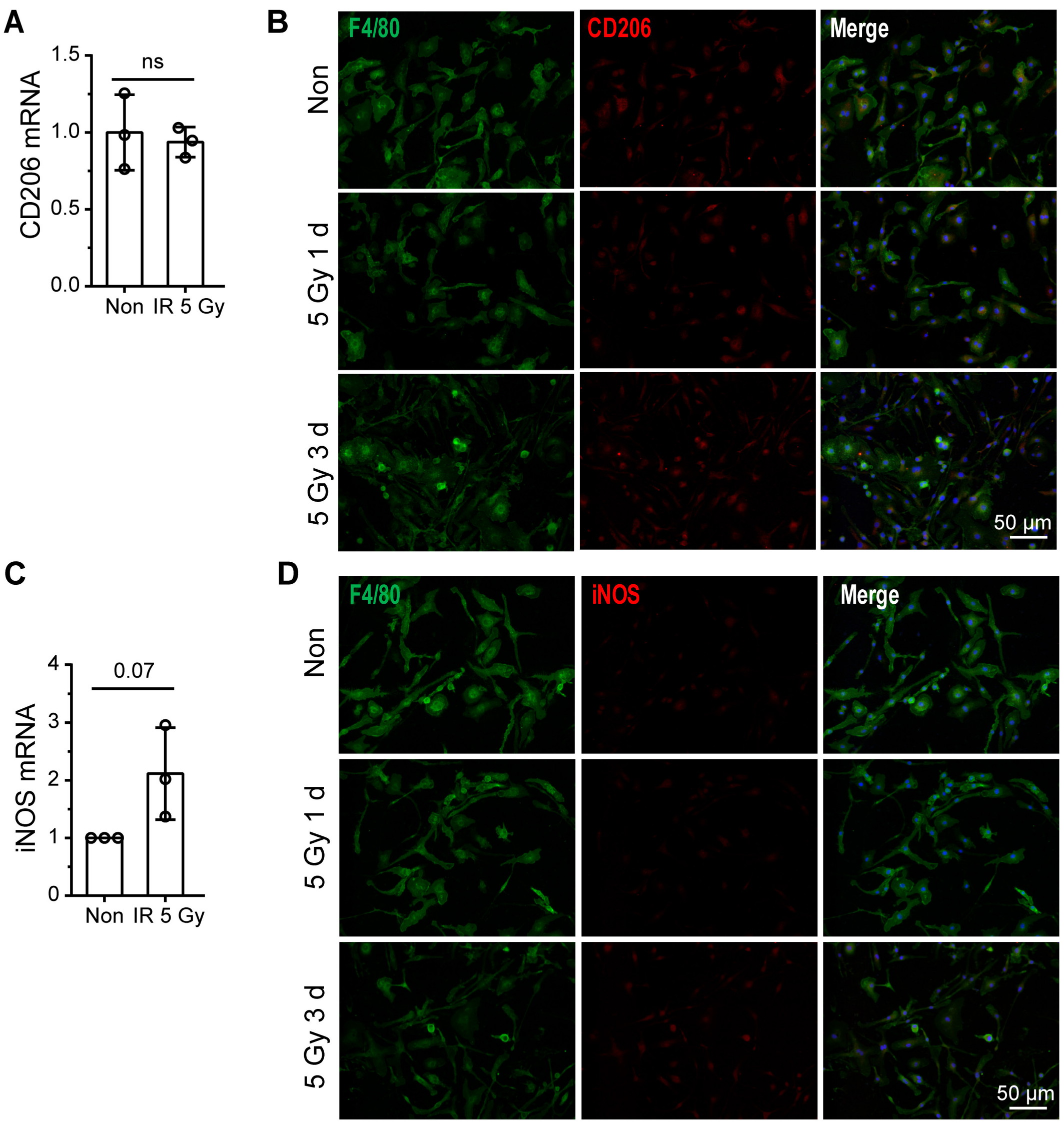


**Figure. S5 Direct effects of irradiation on BMDMs activation. (A)** RT-PCR analysis of CD206 (M2-type Mφ maker) mRNA levels in BMDMs 24 h after irradiation (n = 3 independent experiments, *t*-test). **(B)** Representative immunofluorescence images showing expression of CD206 in BMDMs at 24 h after irradiation. **(C)** RT-PCR analysis of iNOS (M1-type Mφ maker) in BMDMs 24 h after irradiation (n = 3 independent experiments, *t*-test). **(D)** Representative immunofluorescence images showing expression of iNOS in BMDMs at 24 h after irradiation. Scale bar, 50 μm. Data were shown as mean ± SD. ns, not significant (*t*-test).

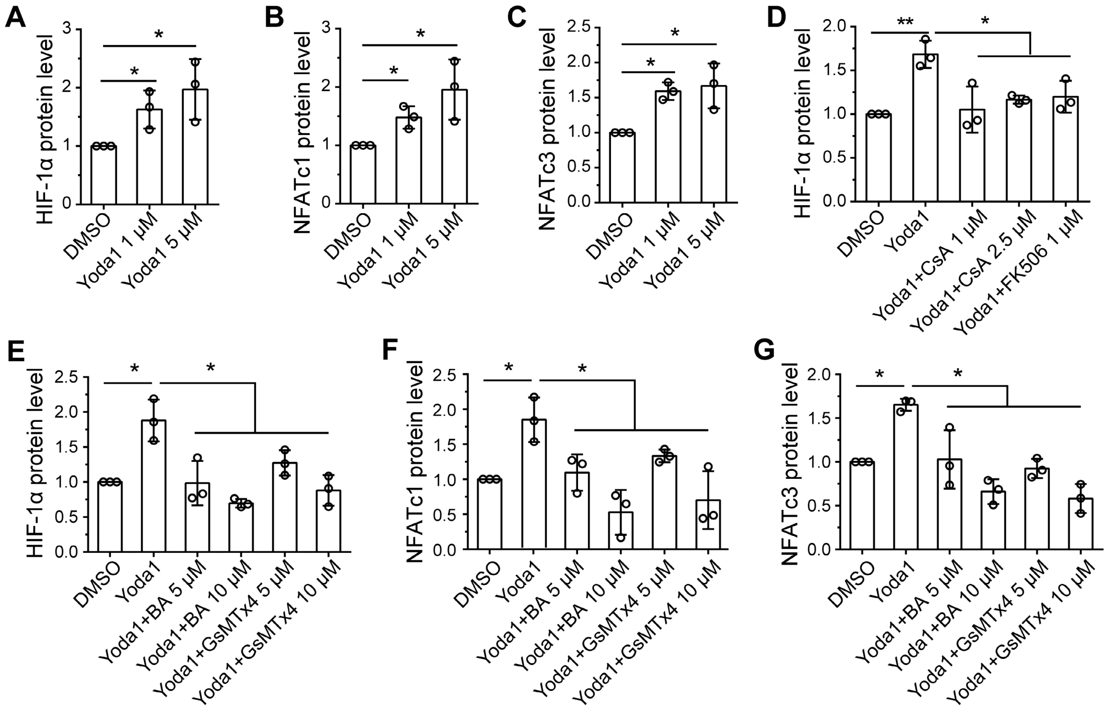


**Figure S6. The quantitative analysis of the relative protein levels of Western blots in Figure 7.** Protein expression was quantified by densitometry and normalized to GAPDH. **(A)** The quantitative analysis of HIF-1α protein level in Fig Figure 7B. **(B-C)** The quantitative analysis of NFATc1 and NFATc3 protein levels in Figure 7E. **(D)** The quantitative analysis of HIF-1α protein level in Figure 7F. **(E-G)** The quantitative analysis of HIF-1α, NFATc1, and NFATc3 protein levels in Figure 7I. n = 3 independent experiments, Tukey test. Data were shown as mean ± SD.*$P < 0.05$; *$P < 0.01$.

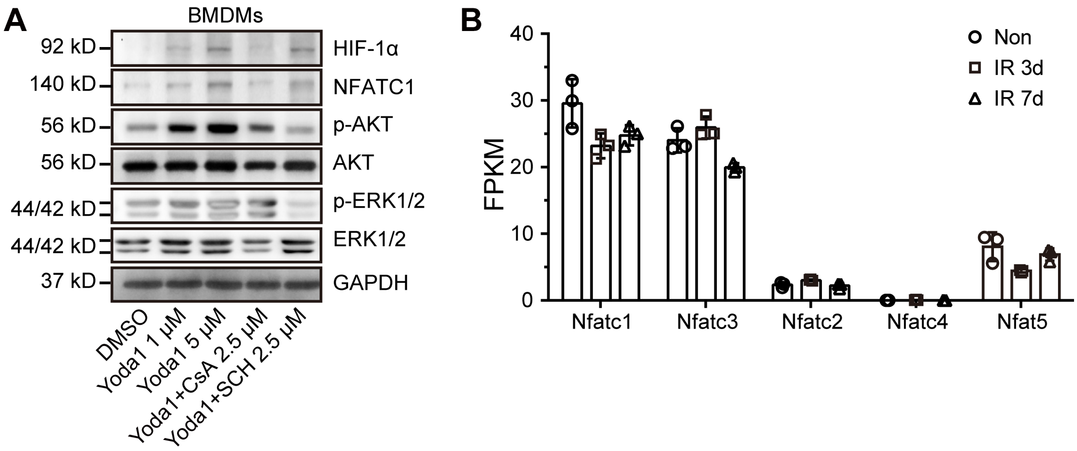


**Figure. S7 The roles of ERK, AKT, and NFATs in Yoda1 induced HIF-1α accumulation. (A)** Western blot analysis of HIF-1α, NFATC1, p-AKT, AKT, p-ERK1/2, ERK, and GAPDH in BMDMs pretreated with CsA or SCH for 30 min before 24 h Yoda1 (5 μM) treatment. Blots are representative of three independent experiments. **(B)** Expression analysis of NFATs in BM-Mφ from mice after 5 Gy irradiation (IR) or non-irradiation (Non) (n = 3 mice). Data were shown as mean ± SD. FPKM: fragments per kilobase million.

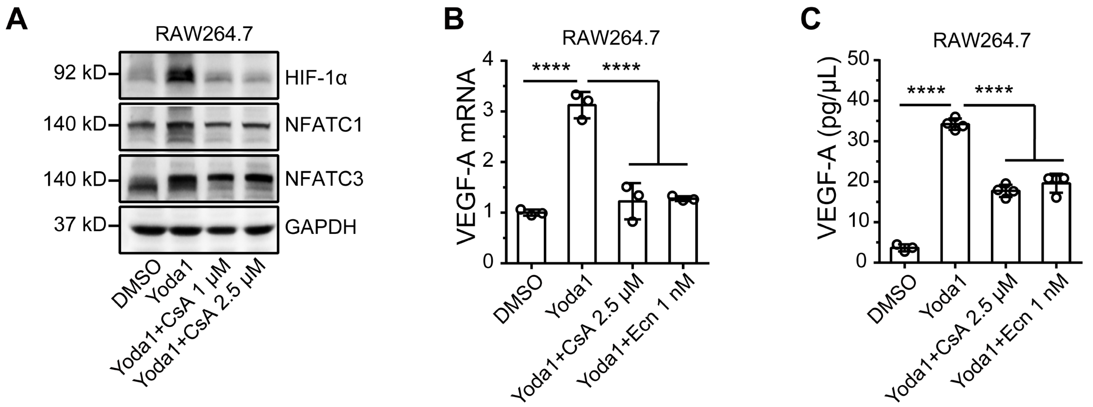


**Figure S8. The activation of calcineurin/NFAT/HIF-1α signaling is responsible for Piezo1-mediated VEGF-A upexpression in RAW264.7 cells. (A)** Western blot analysis of HIF-1α, NFATC1, and NFATC3 in RAW264.7 cells pretreated with CsA for 30 min before 24 h Yoda1 (5 μM) treatment. Blots are representative of three independent experiments. **(B)** RT-PCR analysis of VEGF-A mRNA levels in RAW264.7 cells pretreated with CsA or Ecn for 30 min before 6 h Yoda1 (5 μM) treatment (n = 3 independent experiments, Tukey test). **(C)** ELISA analysis of VEGF-A expression in RAW264.7 cells pretreated with CsA or Ecn for 30 min before 24 h Yoda1 (5 μM) treatment (n = 4 independent experiments, Tukey test). Data were shown as mean ± SD. ****$P < 0.0001$.

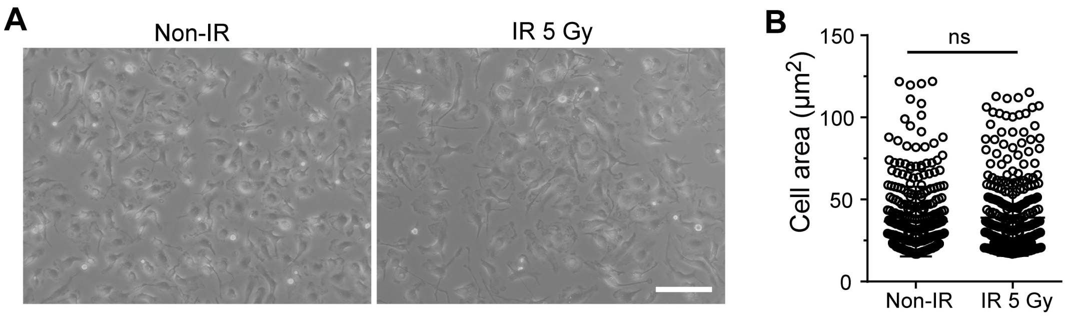


**Figure S9. The morphology of BMDMs after irradiation. (A)** Phase-contrast micrographs of BMDMs on the third day after 5 Gy irradiation. Scale bar, 100 μm. **(B)** Analysis of the cell area of (n = 123, 118 cells from three separate experiments, *t*-test). Values represent mean ± SD, ns, not significant.

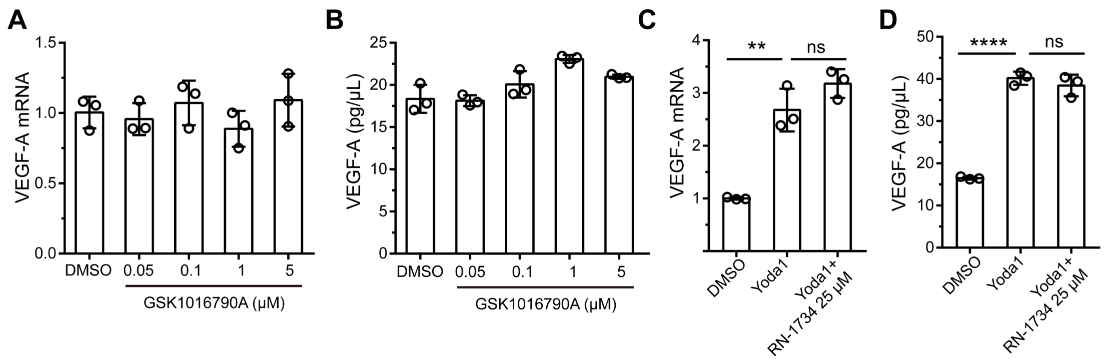


**Figure S10. The role of TRPV4 in the expression of VEGF-A in BMDMs. (A)** RT-PCR analysis of VEGF-A mRNA levels in BMDMs at 6 h after TRPV4 agonist GSK1016790A treatment **(B)** ELISA analysis of VEGF-A expression in BMDMs at 24 h after TRPV4 agonist GSK1016790A treatment. **(C)** RT-PCR analysis of VEGF-A mRNA levels in BMDMs pretreated with TRPV4 inhibitor RN-1734 for 30 min before 6 h Yoda1 (5 μM) treatment **(D)** ELISA analysis of VEGF-A expression in BMDMs pretreated with TRPV4 inhibitor RN-1734 for 30 min before 24 h Yoda1 (5 μM) treatment. n = 3 independent experiments, Tukey test. Data were shown as mean ± SD. $^{**}P < 0.01$; $^{****}P < 0.0001$; ns, not significant.
